# Structure‐guided stabilization of pathogen‐derived peptide‐HLA‐E complexes using non‐natural amino acids conserves native TCR recognition

**DOI:** 10.1002/eji.202149745

**Published:** 2022-02-13

**Authors:** Claire Barber, Victoria Arena De Souza, Rachel L. Paterson, Magdalena Martin‐Urdiroz, Nitha Charles Mulakkal, Velupillai Srikannathasan, Mary Connolly, Gwilym Phillips, Tein Foong‐Leong, Robert Pengelly, Vijaykumar Karuppiah, Tressan Grant, Marcin Dembek, Anil Verma, Dawn Gibbs‐Howe, Thomas H. Blicher, Andrew Knox, Ross A. Robinson, David K. Cole, Sarah Leonard

**Affiliations:** ^1^ Immunocore Ltd Abingdon Oxfordshire UK

**Keywords:** HLA‐E, TCR, non‐natural amino acids, crystal structure, HIV

## Abstract

The nonpolymorphic class Ib molecule, HLA‐E, primarily presents peptides from HLA class Ia leader peptides, providing an inhibitory signal to NK cells via CD94/NKG2 interactions.

Although peptides of pathogenic origin can also be presented by HLA‐E to T cells, the molecular basis underpinning their role in antigen surveillance is largely unknown. Here, we solved a co‐complex crystal structure of a TCR with an HLA‐E presented peptide (pHLA‐E) from bacterial (*Mycobacterium tuberculosis*) origin, and the first TCR‐pHLA‐E complex with a noncanonically presented peptide from viral (HIV) origin. The structures provided a molecular foundation to develop a novel method to introduce cysteine traps using non‐natural amino acid chemistry that stabilized pHLA‐E complexes while maintaining native interface contacts between the TCRs and different pHLA‐E complexes. These pHLA‐E monomers could be used to isolate pHLA‐E‐specific T cells, with obvious utility for studying pHLA‐E restricted T cells, and for the identification of putative therapeutic TCRs.

## Graphical abstract

We show a peptide can be modified using non‐natural amino acids to anchor into the HLA‐E binding groove whilst maintaining native interactions with TCR. This enables the generation of stabilised peptide‐HLA‐E complexes that can be used to isolate and study natural HLA‐E‐restricted T cells in human disease.

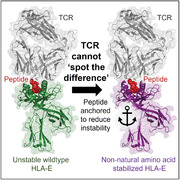

## Introduction

HLA‐E is a nonclassical class Ib HLA expressed by nearly every nucleated cell in the body [1, 2]. Unlike the highly polymorphic HLA class Ia molecules, HLA‐E has two functional alleles, HLA‐E*01:01 and HLA‐E*01:03, which differ by a single amino acid, and are expressed at equal frequencies in the human population [3, 4]. Expression of HLA‐E on the cell surface is usually reliant on binding of leader peptides from HLA‐A, ‐B, ‐C, or ‐G molecules, typically with the sequence VMAPRTL(L/V/I)L [5]. During normal cellular conditions, HLA‐E presented leader peptides [5, 6] prevent lysis by NK cells via binding to inhibitory NK receptors (NKG2A, etc.) [7]. This pathway is utilized by CMV, which expresses a “mimic” leader peptide from the UL40 protein that can be presented by HLA‐E, allowing the virus to avoid immune detection by NK cells [8, 9]. Other emerging evidence indicates a broader role for HLA‐E (and Mamu‐E in primates) in T‐cell pathogen surveillance, demonstrating that HLA‐E‐restricted peptides derived from HIV, hepatitis B virus (HBV), *Mycobacterium tuberculosis* (Mtb), and others can act as ligands for protective T‐cell‐mediated immunity [10, 11, 12, 13, 14].

The structure of HLA‐E is analogous to classical HLA class Ia molecules: it is composed of an α‐chain with three extracellular domains, comprising the α1‐ and α2‐domains that form the peptide binding groove, an α3 domain that associates with β2m, a transmembrane domain, and a cytoplasmic domain [6]. The HLA‐E binding groove is ideally suited to bind leader peptides, or leader “mimic” peptides, that are highly conserved in sequence. In particular, the canonical anchor residues (methionine at position 2 and leucine at the C‐terminal position) play a pivotal role in stabilizing pHLA‐E complexes through specific interactions with the B‐ and F‐pockets in the binding groove [6]. Although HLA‐E can tolerate some other peptide sequences [15], substitution outside of these key residues can have a major impact on the stability of the complex and presentation at the cell surface [10]. This is of relevance for multiple other pathogenic peptides that have been identified as HLA‐E binders [10, 11, 16, 17], many of which differ from the leader sequence motif likely affecting both their presentation mode and stability [10].

Given its broad expression profile, restricted polymorphism, and immunogenic capacity, HLA‐E is an attractive therapeutic target. However, the nature of TCR recognition of pathogen‐derived pHLA‐E complexes is not well established, partly due to the poor stability of some of these complexes [10]. Here, we solved crystal structures of TCRs bound to HLA‐E in complex with well‐characterized peptides from Mtb [11] and HIV [10, 13], demonstrating divergent binding modes akin to the differences observed for TCRs recognizing distinct pHLA class I complexes [18]. Analysis of a range of pHLA‐E complexes confirmed that several pathogen‐derived peptides were of very poor stability, representing a problem for the generation of stable soluble pHLA‐E complexes for the isolation and study of pHLA‐E restricted T cells. Thus, we used our structural analysis as a platform to study both established [19, 20], and novel, pHLA stabilization approaches. We found that, while some established pHLA stabilization approaches perturbed native TCR binding to pHLA‐E, our novel method using non‐natural amino acid (NNAA) chemistry [21], could stabilize pHLA‐E complexes, and maintain native interface contacts and binding affinities between TCRs and different pHLA‐E complexes. This approach was used to generate stable pHLA‐E multimers to isolate pHLA‐E‐specific T cells, representing a unique tool for the characterization of pHLA‐E‐specific T‐cell responses, the isolation of pHLA‐E‐specific TCRs, and the development of pHLA‐E targeted therapies.

## Results

### Pathogen‐derived pHLA‐E complexes can be highly unstable

**Figure 1:**
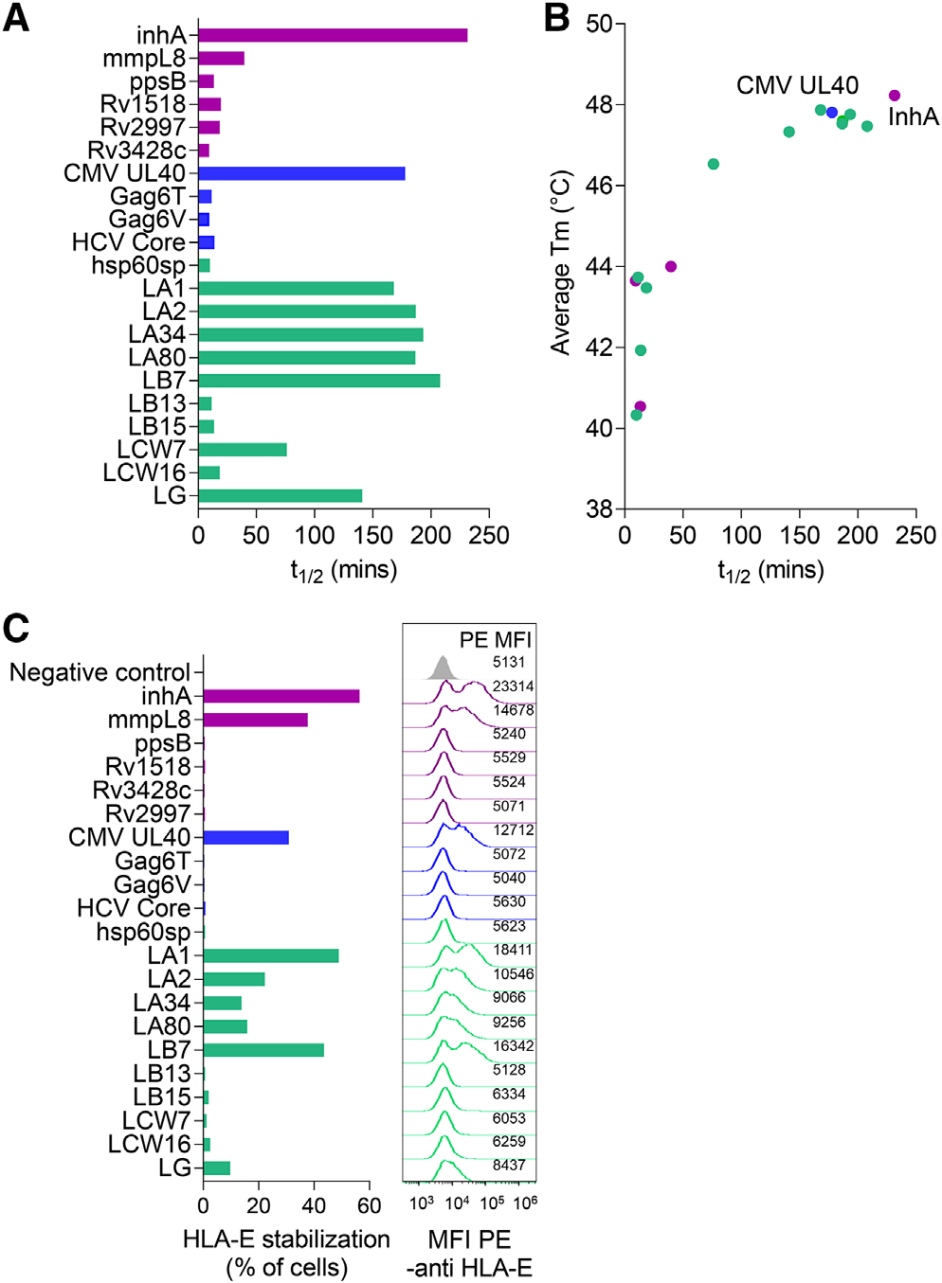
Pathogen‐derived pHLA‐E complexes can be highly unstable. (A) The half‐life (*t*_1/2_) of functionally folded pathogenic and leader peptide loaded HLA‐E as assessed by surface plasmon resonance via detection over time using ILT2. Data representative of at least two independent experiments. (B) Correlation of *t*_1/2_ of pathogenic and leader peptide loaded HLA‐E as assessed by surface plasmon resonance versus average thermal melting point (*Tm*) as assessed by thermal shift assay. Data representative of two independent experiments. (C) Pathogenic and leader peptides binding to HLA‐E as assessed by HLA‐E upregulation at the cell surface by flow cytometry detection using an HLA‐E antibody. Bar graphs display HLA‐E stabilization (% cells in PE gate) with corresponding histograms plots and MFI values of PE‐anti‐HLA‐E staining. The negative control sample shows results for unpulsed cells stained with PE‐anti‐HLA‐E (grey histogram). Gating performed as shown in Supporting information Figure S1. Data representative of two independent experiments. Purple = bacterially derived peptides, blue = virally derived peptides and green = self‐derived peptides.

Several pathogen‐derived peptides have been identified to bind to HLA‐E [10, 11, 16, 17], but their stability has not been comprehensively analyzed in comparison with leader peptides [5]. To evaluate the variation in stability between peptides presented by HLA‐E (HLA‐E*01:03 was used throughout), we performed a comprehensive analysis on 11 HLA class Ia‐derived leader consensus sequences (Supporting information Table S1) and a range of pathogen‐derived peptides (Supporting information Table S2) [9, 10, 11]. The *t*_1/2_ of pHLA‐E complexes was determined by measuring binding of a solubilized HLA‐specific receptor, ILT2, to monitor functionally refolded monomers over time using surface plasmon resonance (SPR) (Fig. 1A, Supporting information Table S2). In addition, we established the thermal melting point (*Tm*) of pHLA‐E complexes using thermal shift assays (Fig. 1B, Supporting information Table S2), and evaluated the cell‐surface expression of pHLA‐E complexes following peptide pulsing as detected by HLA‐E antibodies via flow cytometry (Fig. 1C, Supporting information Fig. S1A).

Overall, pHLA‐E t_1/2_ was highly correlated with *Tm*, and peptides with longer *t*_1/2_ and higher *Tm* had a greater capacity to stabilize HLA‐E on the cell surface (Fig. 1, Supporting information Fig. S1A, Table S2). Of the pathogen‐derived peptides, only inhA_53‐61_ derived from Mtb and UL40_15‐23_ derived from CMV (identical to the sequence of the leader peptide of HLA‐Cw3) were as stable as the most stable HLA class Ia‐leader peptides (*t*_1/2_ = 3.86 h inhA_53‐61_; *t*_1/2_ = 2.96 h UL40_15‐23_). For inhA_53‐61_, relatively good stability was observed despite encoding leucine, rather than methionine, as the primary B‐pocket anchor at peptide position 2. By contrast, all other pathogen‐derived peptides, including the well‐characterized HIV Gag peptides, Gag6T_276‐284_ and the common variant Gag6V_276‐284_ [10, 22], displayed very poor stability binding to HLA‐E (*t*_1/2_ = 0.19 h Gag6T_276‐284_; *t*_1/2_ = 0.16 h Gag6V_276‐284_). However, it should be noted that all of the *t*_1/2_ measurements of pHLA‐E were at least an order of magnitude lower than that observed for most pHLA class Ia complexes (*t*_1/2_ up to 10 h) [23], and 4 of the 10 leader peptides from the HLA class Ia molecules, B13 (LB13_3‐11_), B15 (LB15_3‐11_), Cw7 (LCW7_3‐11_), and Cw16 (LCW16_3‐11_), also demonstrated poor stability in complex with HLA‐E (*t*_1/2_ = 0.19 – 1.27 h).

### Divergent molecular mechanisms underpin TCR recognition of bacterially‐ and virally‐derived pHLA‐E complexes

**Figure 2:**
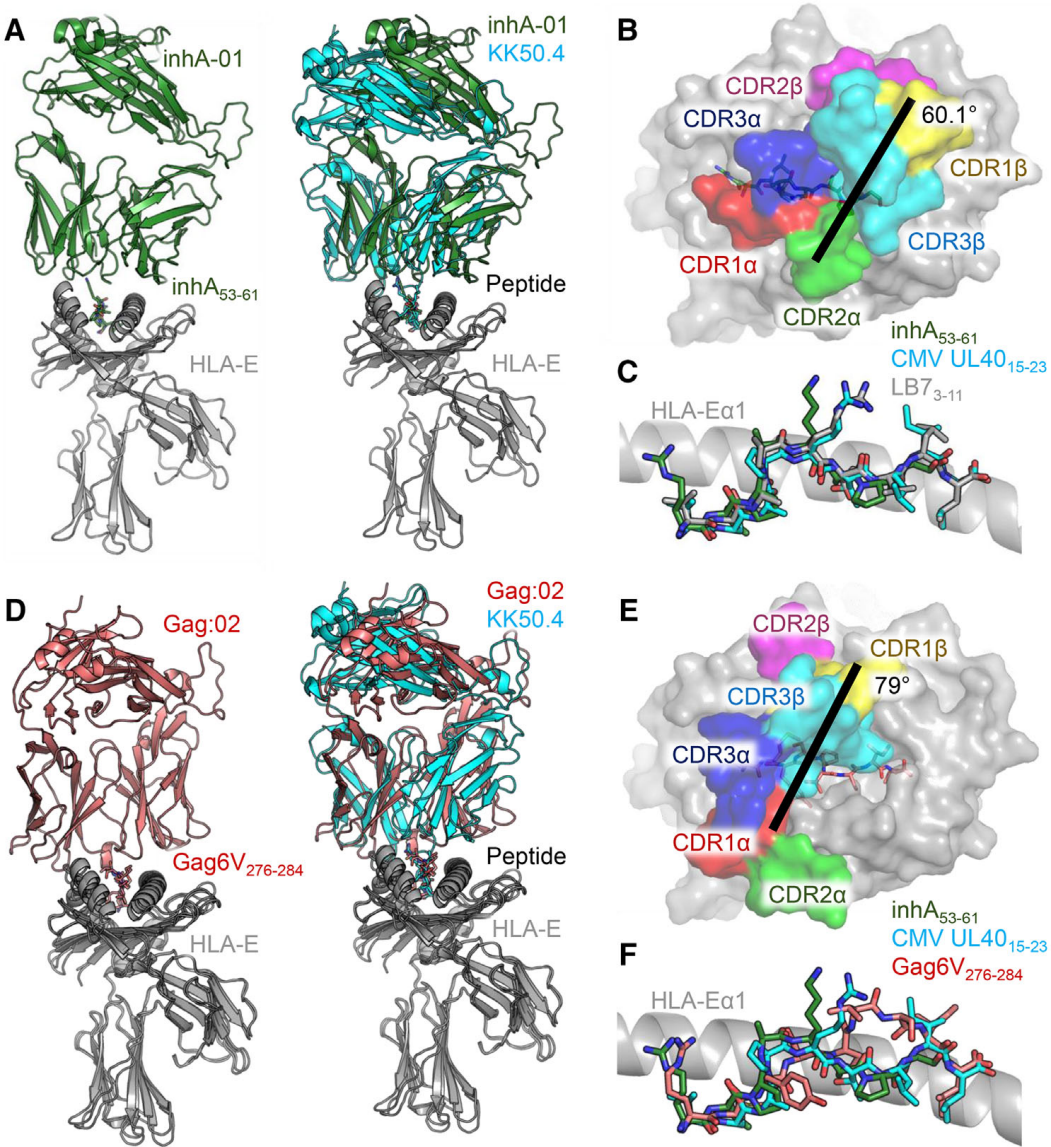
Structural overview of the inhA:01‐HLA‐E‐inhA and Gag:02‐HLA‐E‐Gag6V complexes. (A) LEFT: overview of the inhA:01‐HLA‐E‐inhA complex (inhA:01 TCR shown as green cartoon, HLA‐E shown as gray ribbon, inhA_53‐61_ shown as green sticks). RIGHT: superposition of the inhA:01‐HLA‐E‐inhA complex with the KK50.4‐HLA‐E‐UL40 complex (PDB code: 2ESV) (inhA:01 TCR shown as green cartoon, KK50.4 TCR shown as cyan cartoon, UL40_15‐23_ shown as cyan sticks). (B) Top‐down view of the positioning of the inhA:01 TCR CDR loops (shown as colored surface) over HLA‐E‐inhA. (C) Superposition of HLA‐E bound inhA_53‐61_, CMV UL40_15‐23_ and LB7_3‐11_ (shown as gray sticks, PDB code: 1MHE). (D) LEFT: overview of the Gag:02‐HLA‐E‐Gag6V complex (Gag:02 TCR shown as pink cartoon, Gag6V_276‐284_ shown as pink sticks). RIGHT: superposition of the Gag:02‐HLA‐E‐Gag6V complex with the KK50.4‐HLA‐E‐UL40 complex (PDB code: 2ESV). (E) Top‐down view of the positioning of the Gag:02 TCR CDR loops (shown as colored surface) over HLA‐E‐Gag6V (gray surface with Gag6V_276‐284_ shown as pink sticks). (F) Superposition of HLA‐E bound inhA_53‐61_, CMV UL40_15‐23_ and Gag6V_276‐284_.

We selected several TCRs from TCR phage libraries, constructed using natural TCR‐α and TCR‐β chains isolated from multiple healthy donors that could bind to HLA‐E‐inhA, HLA‐E‐Gag6V, and HLA‐E‐UL40. To provide structural insight into TCR recognition of pathogen‐derived pHLA‐E complexes, and to better understand the nature of TCR binding to very unstable pHLA‐E complexes, we solved the structures of one of these TCRs (inhA:01 TCR) in complex with HLA‐E‐inhA and another (Gag:02 TCR) in complex with the unstable HLA‐E‐Gag6V to 2.26 and 2.55 Å, respectively (Supporting information Table S3). The inhA:01 TCR bound to HLA‐E‐inhA in a conventional mode, with the TCR‐α chain polarized over the HLA‐Eα2 helix and the TCR‐β chain over the HLA‐Eα1 helix, with a crossing angle of 60.1° (Fig. 2A and B). The binding mode was very similar to the co‐complex between the KK50.4 TCR and pHLA‐E (presenting an identical peptide derived from both the leader peptide LCW3_3‐11_ and CMV UL40_15‐23_, hereafter referred to as HLA‐E‐UL40) [9]. The inhA_53‐61_ peptide in the inhA:01‐HLA‐E‐inhA complex adopted a virtually identical conformation to UL40_15‐23_ peptide in the KK50.4‐HLA‐E‐UL40 complex [9] and the HLA‐B7 class Ia leader peptide in complex with HLA‐E (HLA‐E‐LB7) [6], forming a conserved central bulge at peptide residues 4 and 5 (Fig. 2C). The Gag:02 TCR also bound to HLA‐E‐Gag6V in a conventional overall mode compared to most other TCR‐pHLA complexes, with a crossing angle of 79° (Fig. 2D and E). However, the Gag6V_276‐284_ peptide adopted a distinct non‐canonical conformation, compared to inhA_53‐61_ and UL40_15‐23_, in the complex structures, forming a bulge between residues 6–8, consistent with the previously published apo structure of HLA‐E‐Gag6V [10] (Fig. 2F).

**Figure 3:**
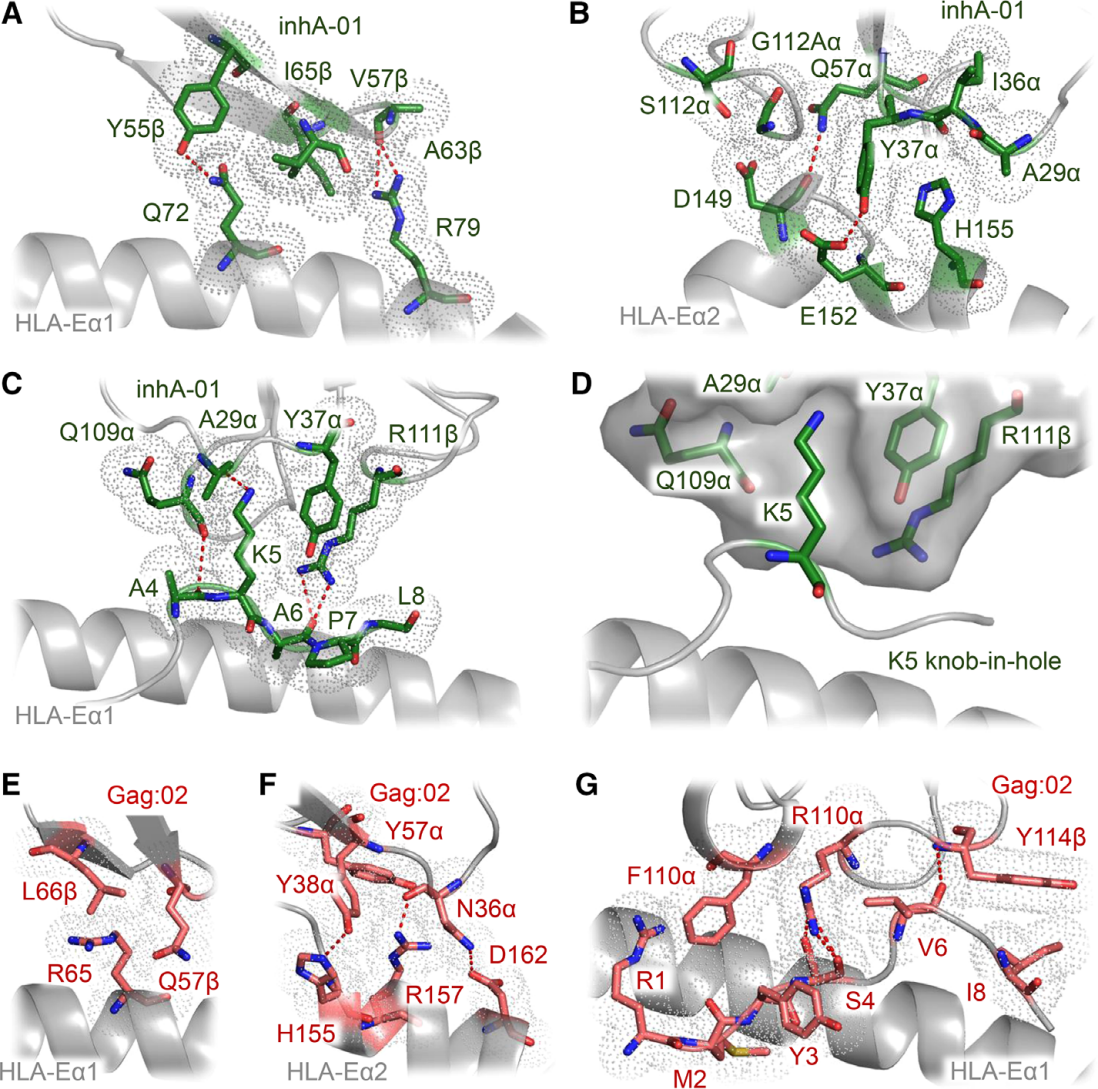
Key interactions in the inhA:01‐HLA‐E‐inhA and Gag:02‐HLA‐E‐Gag6V complexes. (A) Main inhA:01 TCR interactions with HLA‐Eα1 (green sticks). (B) Main inhA:01 TCR interactions with HLA‐Eα2 (green sticks). (C) inhA:01 TCR interactions with inhA_53‐61_ (green sticks). (D) Surface (gray) representation of the pocket formed by the inhA:01 TCR residues A30, Y32, Q96 and R98 (green sticks) interacting with inhA_53‐61_ residue K5 (green sticks). (E) Main Gag:02 TCR interactions with HLA‐Eα1 (pink sticks). (F) Main Gag:02 TCR interactions with HLA‐Eα2 (pink sticks). (G) Gag:02 TCR interactions with Gag6V_276‐284_ (pink sticks). Hydrogen bonds (<3.4Å) are shown as red dotted lines. Gray dots indicate sphere of vdW (<4Å) interactions.

Further analysis of the main contact zones between each TCR‐pHLA‐E complex demonstrated that the inhA:01 TCR was stabilized via interactions focused on HLA‐E residues Q72, R79, D149, E152, and H155 (Fig. 3A and B), positioning residues in the TCR CDR‐1α, CDR‐3α, and CDR‐3β to make key contacts with inhA_53‐61_ peptide residues A4, K5, A6, P7, and L8 (Fig. 3C). The main interaction with the inhA_53‐61_ peptide was mediated by a pocket formed by inhA:01 TCR residues A29α, Y37α, Q109α, and R111β, enabling a knob‐in‐hole like interaction with inhA_53‐61_ peptide residue K5 (Fig. 3D).

The Gag:02 TCR utilized a different interaction mechanism compared to both KK50.4 and inhA:01 TCRs (both of which made very focused interactions primarily with a single peptide residue), predominantly engaging HLA‐E residues R65, H155, R157 and D162 (Fig. 3E and F) and positioning residues in the TCR CDR3α and CDR3β to make a much broader and balanced set of interactions with Gag6V_276‐284_ peptide residues R1, M2, Y3, S4, V6, and I8 (Fig. 3G). This binding mode was partly directed by the noncanonical conformation of the Gag6V_276‐284_ peptide, which formed a bulge that was shifted toward the C‐terminus of the peptide, compared to the more central location observed for HLA‐E‐inhA, and previously published pHLA‐E‐leader complexes [6, 9, 10]. Combined with the recent structure of the GF4 TCR in complex with HLA‐E‐UL40, in which the GF4 TCR bound with a unique binding mode compared to the KK50.4 TCR [24], these findings suggest that TCRs that recognize pHLA‐E can use divergent binding modes, mirroring classical TCR‐pHLA‐I complexes [18, 25, 26].

### Conventional cysteine traps can stabilize pHLA‐E complexes, but modulate native TCR recognition

**Figure 4:**
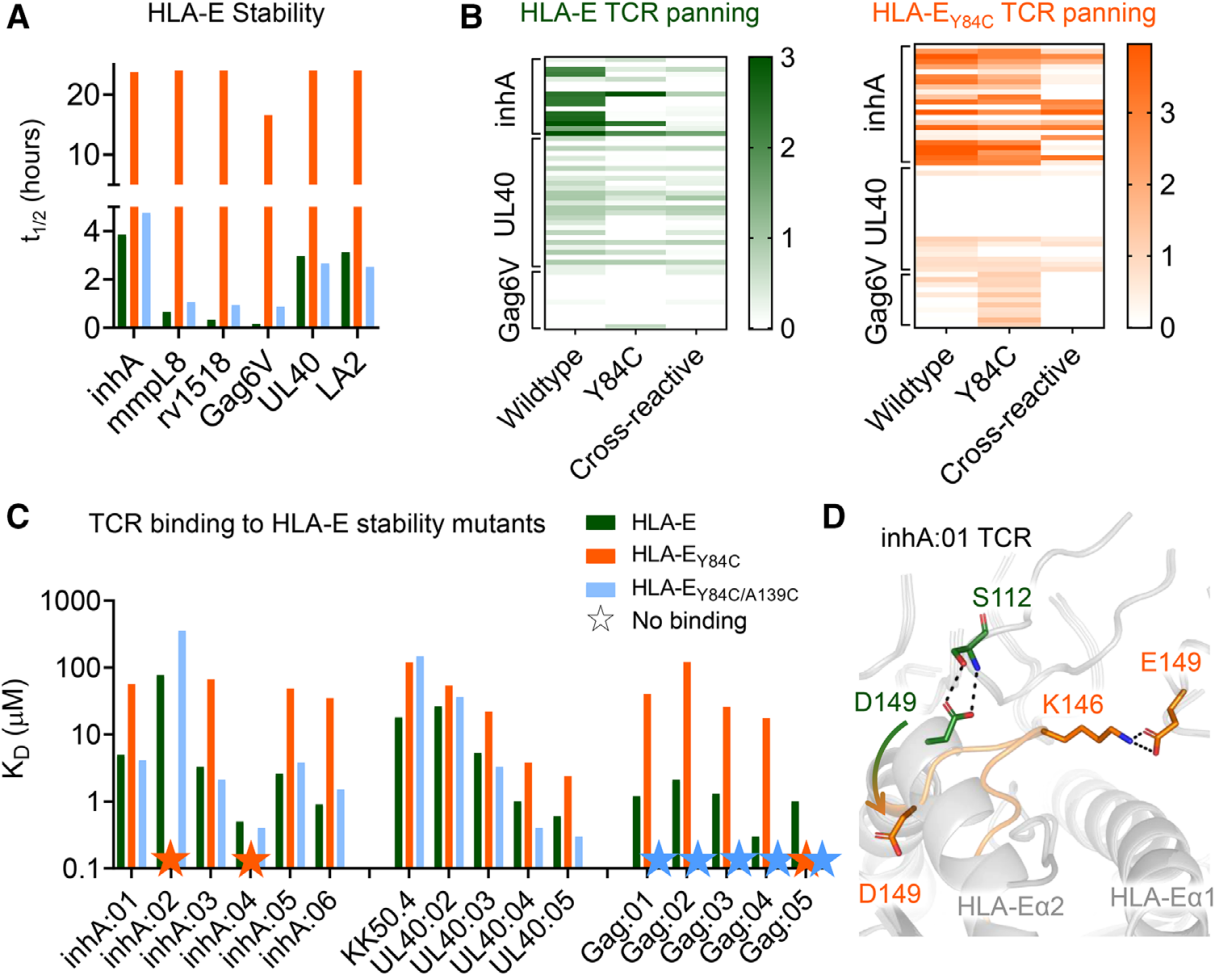
Characterization of stability of, and TCR binding to, HLA‐E with conventional cysteine trapped pHLA. (A) The *t*_1/2_ of pathogenic and leader peptide‐loaded HLA‐E as assessed by surface plasmon resonance. WT pHLA‐E shown as green bars, pHLA‐E_Y84C_ shown as orange bars, and HLA‐E_Y84C/A139C_ shown as blue bars. Data representative of two independent experiments. (B) Heat map of ELISA results from inhA, UL40, or Gag6V phage biopanning outputs using WT HLA‐E (green), or HLA‐E_Y84C_ (orange). Clones are arranged horizontally across the heat map and are grouped along the *y*‐axis according to which HLA‐E target complex they were panned against. Antigens tested by ELISA are indicted along the *x*‐axis; Wildtype = unmodified pHLA‐E. Y84C = HLA‐E_Y84C_peptide. Cross‐reactive = TCRs that bind to a mixture of leader peptides presented by HLA‐E. (C) TCR binding affinity, as assessed by surface plasmon resonance, depicting the equilibrium dissociation constant (*K_D_*) of the WT pHLA‐E (green bars) and the cysteine trapped pHLA variants (pHLA‐E_Y84C_–orange bars and pHLA‐E_Y84C/A139C_–blue bars) for multiple different TCRs recognizing inhA, UL40 and Gag6V peptides. Colored stars = no binding detected. Data representative of at least two independent experiments. (D) Superposition of the inhA:01 TCR in complex with WT HLA‐E‐inhA, or HLA‐E_Y84C_‐inhA. Key differences in TCR contacts between wildtype HLA‐E‐inhA and HLA‐E_Y84C_‐inhA are shown in green sticks and orange sticks, respectively. The arrow shows the flipped orientation of D149 in the inhA:01‐HLA‐E_Y84C_‐inhA complex.

Our new co‐complex structures of the inhA:01 and Gag6V:02 TCRs, combined with the previously published KK50.4 TCR [9], provided a strong molecular foundation for the characterization and design of strategies to stabilize unstable pathogen‐derived pHLA‐E complexes while maintaining the fidelity of the native TCR‐pHLA‐E binding mode. This is a particularly important consideration for the development of pHLA‐E multimers for the isolation and characterization of native pHLA‐E reactive T cells, as well as for the selection of specific TCRs as tools, or potential therapeutics. Multiple pHLA‐stabilization approaches have been previously reported for HLA class Ia molecules, which involve engineering the HLA by either introducing a single (Y84C) or double mutation (Y84C/A139C) into the heavy chain of HLA. To increase the stability of peptide bound to the Y84C mutant, an additional cysteine is introduced at the C‐terminal end of the peptide (+GC or +GCG), resulting in a disulfide linkage to trap the peptide. In contrast, the Y84C/A139C mutant stabilizes the HLA groove itself without the need for peptide modification [19, 20]. We applied both these approaches to HLA‐E using a selection of pathogen‐ and leader‐derived peptides (Fig. 4A, Supporting information Table S4) and found that, while the HLA‐E_Y84C_ modification substantially increased the *t*_1/2_ of pHLA‐E for all peptides tested (*t*_1/2_ = 16.6 – >24 h), the HLA‐E_Y84C/A139C_ modification resulted in only modest improvements in pHLA‐E stability (*t*_1/2_ = 0.88–4.75 h).

Having established that the HLA‐E_Y84C_ mutation stabilized pHLA‐E complexes, we next investigated whether this approach could be utilized for discovery of antigen‐specific TCRs using phage panning of human TCR libraries as previously reported [27]. TCR variants were isolated by biopanning against WT HLA‐E or HLA‐E_Y84C_, in complex with inhA_53‐61_, UL40_15‐23_, or Gag6V_276‐284_ peptides. Overall, each phage biopanning condition generated approximately equal numbers of productive clones, defined as clones lacking stop codons or frame shifts in the TCR sequence, except for the WT HLA‐E‐Gag6V, which generated a higher proportion of unproductive clones (Supporting information Fig. S2A). Panning on HLA‐E‐inhA and HLA‐E_Y84C_‐inhA resulted in some shared clones (i.e., clones that shared an identical αβ‐TCR sequence). Likewise, some shared clones were derived from panning on HLA‐E‐UL40 and HLA‐E_Y84C_‐UL40. In contrast, none of the clones isolated from panning on HLA‐Gag6V and HLA‐E_Y84C_‐Gag6V were shared (Supporting information Fig. S2B).

The clones were next assessed for their ability to recognize their respective target peptide presented in the context of either the WT pHLA‐E or pHLA‐E_Y84C_. Clones were also tested for cross‐reactivity against a mixture of leader peptides presented by HLA‐E (HLA‐E‐leader). The leader peptide CW3_3‐11_, which has an identical sequence to CMV UL40_15‐23_, was not used in the cross‐reactivity panel, where HLA‐E‐UL40 was the target. Approximately 30% of TCRs isolated by panning on either HLA‐E‐inhA or HLA‐E‐UL40 were specific for their respective HLA‐E‐target peptide complexes, with around 50% of the TCRs demonstrating cross‐reactivity with HLA‐E‐leader and approximately 20% exhibiting no binding to any HLA‐E peptide complex tested (Supporting information Fig. S2C). In contrast, none of the TCR clones isolated from panning on HLA‐E‐Gag6V exhibited specific binding to HLA‐E‐Gag6V; most clones also failed to recognize HLA‐E‐leader. Panning on HLA‐E_Y84C_‐peptide complexes resulted in ∼10% TCRs specific for HLA‐E‐UL40, approximately 29% specific for HLA‐E‐inhA and around 60% that were HLA‐E‐Gag6V specific (Supporting information Fig. S2C). This finding is consistent with a recent publication demonstrating that HLA‐E_Y84C_ could be used to isolate HLA‐E‐Gag6V‐specific TCRs from patient blood, providing additional evidence that this epitope is physiologically relevant [13]. Although the absolute numbers suggest that HLA‐E_Y84C_ could be used to isolate HLA‐E‐inhA and HLA‐E‐Gag6V‐specific TCRs, further inspection of the ELISA data demonstrated very different patterns in terms of the strength of the signal when comparing reactivity to WT HLA‐E, possibly directed by modified TCR recognition (Fig. 4B).

Although the Y84C and Y84C/A139C mutations are distal to the central portion of the peptide that usually comprises the main TCR contact zone, previous reports with HLA class Ia molecules have shown that similar stabilizing modifications can alter TCR binding [28]. Thus, we tested the binding affinity of our panel of TCRs (selected from TCR libraries using HLA‐E‐inhA, HLA‐E‐Gag6V and HLA‐E‐UL40) against their cognate peptides bound to WT HLA‐E, HLA‐E_Y84C_, and HLA‐E_Y84C/A139C_ using SPR (Fig. 4C, Supporting information Fig. S3). In most cases, TCR binding affinity was substantially altered, and in many cases abrogated, using the engineered HLA‐E‐peptide variants. To gain structural insights into this observation, we solved the crystal structure of the inhA:01 TCR in complex with HLA‐E_Y84C_‐inhA (Supporting information Table S3, Fig. S4). The binding mode of the inhA:01‐HLA‐E_Y84C_‐inhA complex appeared altered compared to the inhA:01‐HLA‐E‐inhA complex, likely due to the α‐helix formed by residues 140–150 in the WT HLA‐E α2 domain adopting an elongated state in HLA‐E_Y84C_ (Fig. 4D). This led to HLA‐E residue D149 flipping down, abrogating an interaction with inhA:01 TCR residue S112, and alterations of the interaction interface toward the C‐terminal end of the inhA_53‐61_ peptide (Fig. 4D). Thus, the fidelity of TCR binding to these engineered HLA‐E variants was not maintained, providing a likely explanation for the modified binding affinities, and ELISA results, compared to WT HLA‐E.

### Non‐natural amino acid stabilization of pHLA‐E complexes maintains native TCR recognition

**Figure 5:**
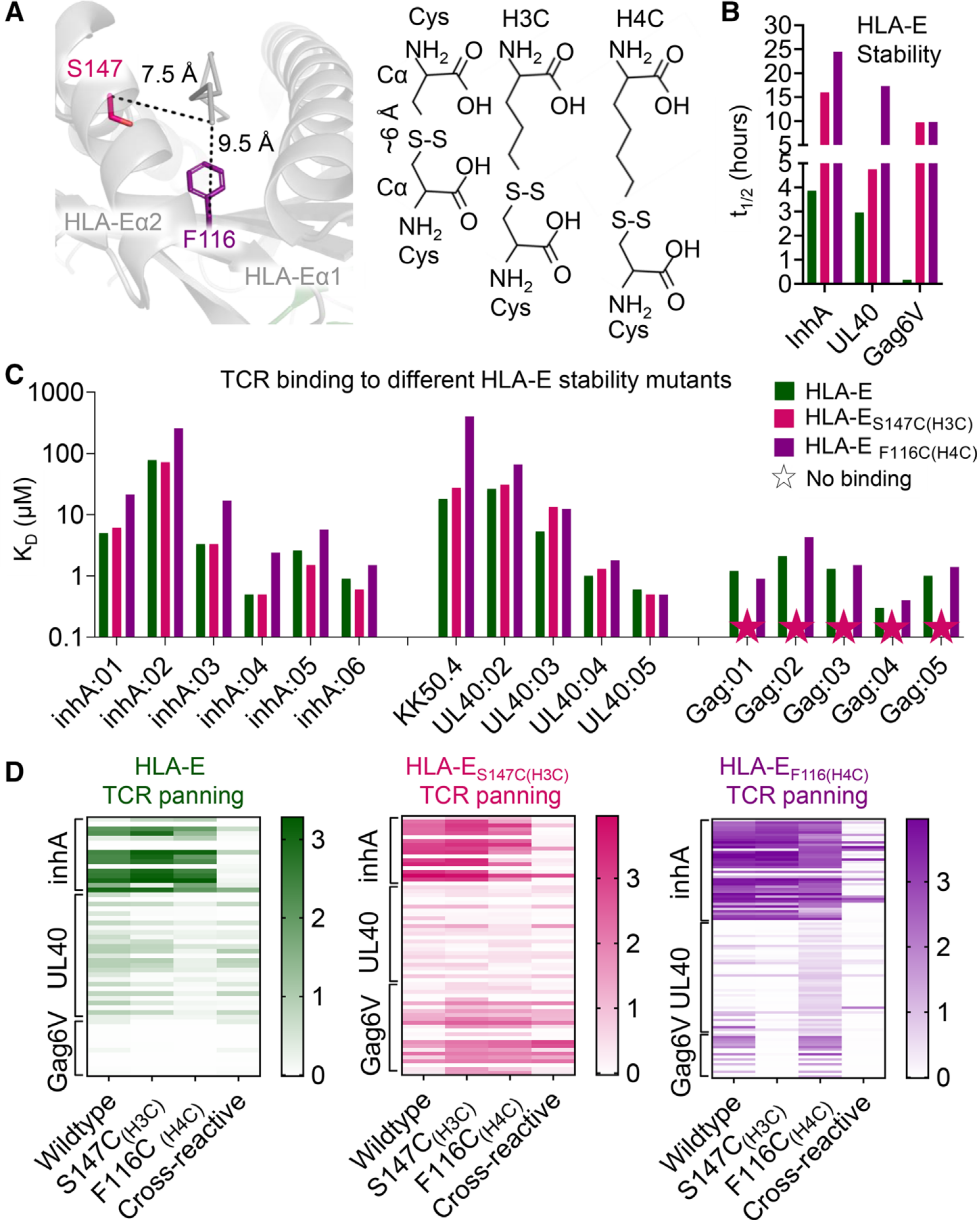
Characterization of stability of, and TCR binding to, HLA‐E with non‐natural amino acid trapped peptides. (A) LEFT: Wildtype apo pHLA‐E (gray cartoon) with the residues for non‐natural amino acid cysteine trapping drawn as colored sticks. RIGHT: Representation of distance between Cα positions in disulfide bonded pair of cysteine residues compared to non‐natural cysteine‐like amino acids with additional methylene groups. (B) The *t*_1/2_ of inhA, UL40, and Gag6V peptide‐loaded HLA‐E. WT pHLA‐E shown as green bars, HLA‐E_S147C(H3C)_peptide shown as pink bars, and HLA‐E_F116C(H4C)_peptide shown as purple bars. Data representative of two independent experiments. (C) TCR binding affinity, as assessed by surface plasmon resonance, depicting equilibrium dissociation constant (*K_D_*) of the WT pHLA‐E (green bars) and the non‐natural amino acid trapped peptide variants (pHLA‐E_S147C(H3C)_peptide – pink bars and pHLA‐E_F116C(H4C)_peptide – purple bars) for multiple different TCRs recognizing inhA, UL40, and Gag6V peptides. Colored stars = no binding detected. Data representative of at least two independent experiments. (D) Heat map of ELISA results from inhA, UL40, or Gag6V phage biopanning outputs using WT HLA‐E (green), HLA‐E_S147C(H3C)_peptide (pink), or HLA‐E_F116C(H4C)_peptide (purple). Clones are arranged horizontally across the heat map and are grouped along the y‐axis according to which HLA‐E target complex they were panned against. Antigens tested by ELISA are indicted along the x‐axis; Wildtype = unmodified pHLA‐E, S147C_(H3C)_ = S147C_(H3C)_peptide, F116C_(H4C)_ = F116C_(H4C)_peptide. Cross‐reactive = TCRs that bind to a mixture of leader peptides presented by HLA‐E.

We used our co‐complex structures and structural modeling to identify alternative stabilizing mutations in HLA‐E that might not modulate TCR recognition. This structural analysis suggested that several HLA‐E residues, including S147 and F116, were in proximity to the C‐terminus of the peptide, but the distances were not optimal for cysteine trapping without potentially altering the dynamics of the complex. As NNAA chemistry can be used to fine tune residues to optimize molecular distancing in engineered proteins, we modified each C‐terminal peptide residue with NNAAs containing additional methylene groups, linked to a sulfanyl moiety, to provide a tailored bridge between the peptide and the HLA‐E binding groove (Fig. 5A). This NNAA approach maintained the native peptide length, unlike for Y84C that required additional residues at the C‐terminal end of the peptide.

Several versions of NNAA‐modified peptides were screened in complex with HLA‐E engineered to encode cysteines at different positions in the peptide binding groove (data not shown). Of these, HLA‐E_S147C_ with peptides modified with the H3C NNAA (HLA‐E_S147C(H3C)_peptide) provided similar benefits to HLA‐E_Y84C_ in terms of stabilizing pHLA‐E complexes (*t*_1/2_ = 4.75– > 24 h) (Fig. 5B, Supporting information Table S4), but did not substantially affect the binding affinity of different TCRs recognizing HLA‐E‐inhA and HLA‐E‐UL40 (Fig. 5C, Supporting information Fig. S5). Although this mutation did abrogate the binding of five HLA‐E‐Gag6V‐specific TCRs, we identified an alternative mutation, HLA‐E_F116C_ with peptides modified with the H4C NNAA (HLA‐E_F116C(H4C)_peptide), that provided similar benefits to HLA‐E_S147C(H3C)_peptide in terms of stabilizing pHLA‐E complexes (HLA‐E‐Gag6V *t*_1/2_ = 0.16 h, HLA‐ E_F116C(H4C)_Gag6V *t*_1/2_ = 9.83 h) (Fig. 5B, Supporting information Table S4), but did not substantially affect the binding affinity of the HLA‐E‐inhA, HLA‐E‐UL40, or HLA‐E‐Gag6V‐specific TCRs (Fig. 5C, Supporting information Fig. S5).

Having established that these mutations could stabilize pHLA‐E complexes without substantially altering TCR binding affinity, we next investigated whether HLA‐E‐peptide complexes stabilized through incorporation of NNAA could be utilized to select antigen‐specific TCRs from human TCR libraries. TCR libraries were constructed using natural TCR‐α and TCR‐β chains isolated from multiple healthy donors expressed as native TCR‐αβ complexes on phage particles. TCR variants were isolated by biopanning against WT HLA‐E, HLA‐E_S147C(H3C)_peptide, or HLA‐E_F116C(H4C)_peptide in complex with inhA_53‐61_, UL40_15‐23_, or Gag6V_276‐284_ peptides. Both HLA‐E_S147C(H3C)_peptide and HLA‐E_F116C(H4C)_peptide generated similar or greater numbers of productive clones (i.e., clones encoding an αβ‐TCR with no stop codons, etc.) compared to WT HLA‐E (Supporting information Fig. S2A). For the inhA_53‐61_ and UL40_15‐23_ peptides, HLA‐E, HLA‐E_S147C(H3C)_peptide, and HLA‐E_F116C(H4C)_peptide produced some shared clones, while for the Gag6V_276‐284_ peptide, no clones were shared (Supporting information Fig. S2B). The clones were next assessed by ELISA for their ability to bind to their respective target peptide presented in the context of either the WT HLA‐E, HLA‐E_S147C(H3C)_peptide, or HLA‐E_F116C(H4C)_peptide, as well as the HLA‐E‐leader peptide cross‐reactivity mix. For the HLA‐E_S147C(H3C)_peptide and HLA‐E_F116C(H4C)_peptide panning, between 20 and 40% clones encoding productive TCRs were specific for either HLA‐E‐inhA or HLA‐E‐UL40. For HLA‐E‐Gag6V, in line with the TCR binding affinity data (Fig. 5C and Supporting information Fig. S5), HLA‐E_S147C(H3C)_Gag6V only generated ∼8% clones encoding a HLA‐E‐Gag6V‐specific TCR, while HLA‐E_F116C(H4C)_Gag6V generated ∼60% (Supporting information Fig. S2C). Importantly, in contrast with the HLA‐E_Y84C_ panning (Fig. 4B), ELISA data demonstrated more similar patterns in terms of the strength of the signal generated by the TCRs encoded by each clone selected using the WT HLA‐E versus HLA‐E_S147C(H3C)_peptide or HLA‐E_F116C(H4C)_peptide (Fig. 5D), in agreement with the TCR binding affinity data (Fig. 5C and Supporting information Fig. S5).

### Structural basis for TCR recognition of non‐natural amino acid stabilized pHLA‐E complexes

To further elaborate on the molecular mechanisms governing the native‐like recognition of our NNAA stabilized pHLA‐E complexes, we solved the co‐complex crystal structures of the inhA:01 TCR in complex with both HLA‐E_S147C(H3C)_inhA and HLA‐E_F116C(H4C)_inhA, the KK50.4 TCR with HLA‐E_F116C(H4C)_UL40, and the Gag6V:02 TCR in complex with HLA‐E_F116C(H4C)_Gag6V (Supporting information Fig. S4, Table S3). In contrast to HLA‐E_Y84C_ co‐complex (Fig. 4D), the inhA:01 TCR‐HLA‐E_S147C(H3C)_inhA crystal structure demonstrated that the native secondary structure of the HLA‐E α2 helix was maintained (Supporting information Fig. S6A). The modification did not alter the overall conformation of the inhA_53‐61_ peptide (Supporting information Fig. S6B), enabling the inhA:01 TCR to make a virtually identical network of interactions with both the HLA‐E surface and bound peptide (Supporting information Fig. S6C). Although the stabilization experiments clearly demonstrated an enhanced effect of this mutation, we were unable to robustly detect the C147‐mediated disulfide bridge either in the crystal structure, or by mass spectrometry (MS) experiments (Supporting information Fig. S7).

**Figure 6:**
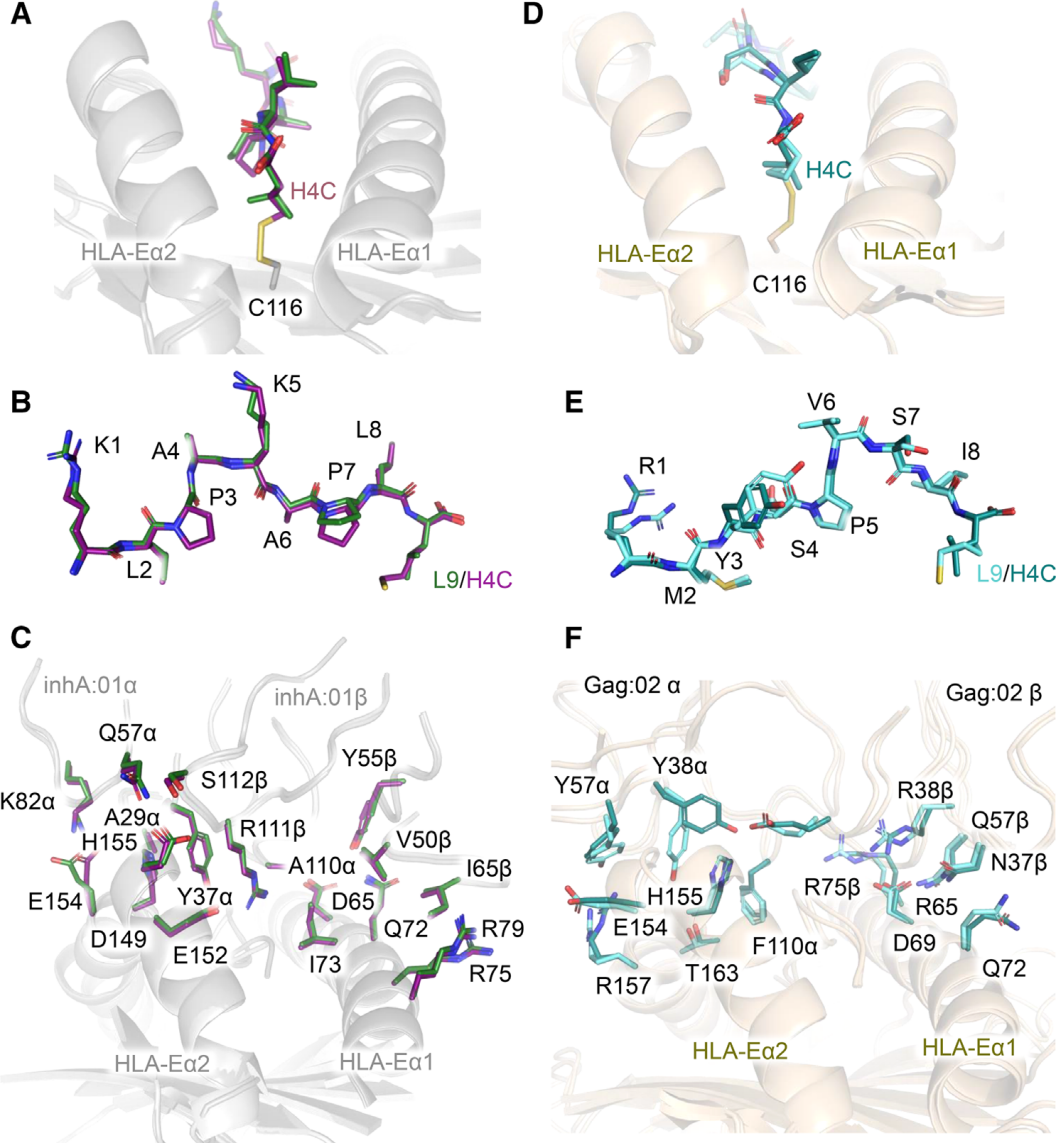
Structural comparison of HLA‐E‐inhA‐ and HLA‐E‐Gag6V‐specific TCRs bound to their respective WT pHLA‐E and pHLA‐E_F116C(H4C)_peptide complexes. (A‐C) Superposition of the WT inhA:01‐HLA‐E‐inhA complex and inhA:01‐HLA‐E_F116C(H4C)_inhA complex calculated considering the HLA chain only. (A) HLA‐E‐inhA and HLA‐E_F116C(H4C)_inhA with HLA‐E shown in gray ribbon, inhA peptide shown in dark green sticks, and HLA‐E_F116C_ shown in gray ribbon with C116 as sticks, H4C inhA‐modified peptide shown in purple sticks. (B) inhA peptide presented by WT HLA‐E, and HLA‐E_F116C(H4C)_inhA. (C) inhA:01 TCR in complex with WT HLA‐E‐inhA or HLA‐E_F116C(H4C)_inhA showing key TCR‐pHLA interface contacts between WT HLA‐E‐inhA and HLA‐E_F116C(H4C)_inhA. (D‐F) Superposition of the WT Gag:02‐HLA‐E‐Gag6V complex and Gag:02‐HLA‐E_F116C(H4C)_Gag6V complex calculated considering the HLA chain only. (D) HLA‐E‐Gag6V and HLA‐E_F116C(H4C)_Gag6V with HLA‐E shown in wheat ribbon, H4C Gag6V_276‐284_ peptide shown in deep teal sticks, and HLA‐E_F116C_ shown in wheat ribbon with C116 as sticks, Gag6V_276‐284‐_modified peptide shown in aquamarine sticks. (E) Wildtype HLA‐E‐Gag6V, and HLA‐E_F116C(H4C)_Gag6V. (F) Gag:02 TCR in complex with WT HLA‐E‐Gag6V or HLA‐E_F116C(H4C)_inhA showing key TCR‐pHLA interface contacts between WT HLA‐E‐Gag6V and HLA‐E_F116C(H4C)_Gag6V.

The crystal structures of the inhA:01, KK50.4, and Gag:02 TCRs in complex with HLA‐E_F116C(H4C)_peptide confirmed that the NNAA modification stabilized these complexes while maintaining the native secondary structure of the HLA‐Eα2 helix (Fig. 6 and Supporting information Fig S8). Clear electron density for the disulfide bond between F116C and the NNAA‐modified peptides was observed (Supporting information Fig. S4), and this was corroborated by MS analysis showing that the covalently bound peptide was the predominant species (Supporting information Fig S7). The modification did not alter the overall conformation of the any of the three peptides tested, and the interaction interfaces between the TCRs and the pHLA‐E surfaces were indistinguishable from the WT co‐complexes.

### Non‐natural amino acid stabilized pHLA‐E multimers enable isolation of antigen‐specific T cells

**Figure 7:**
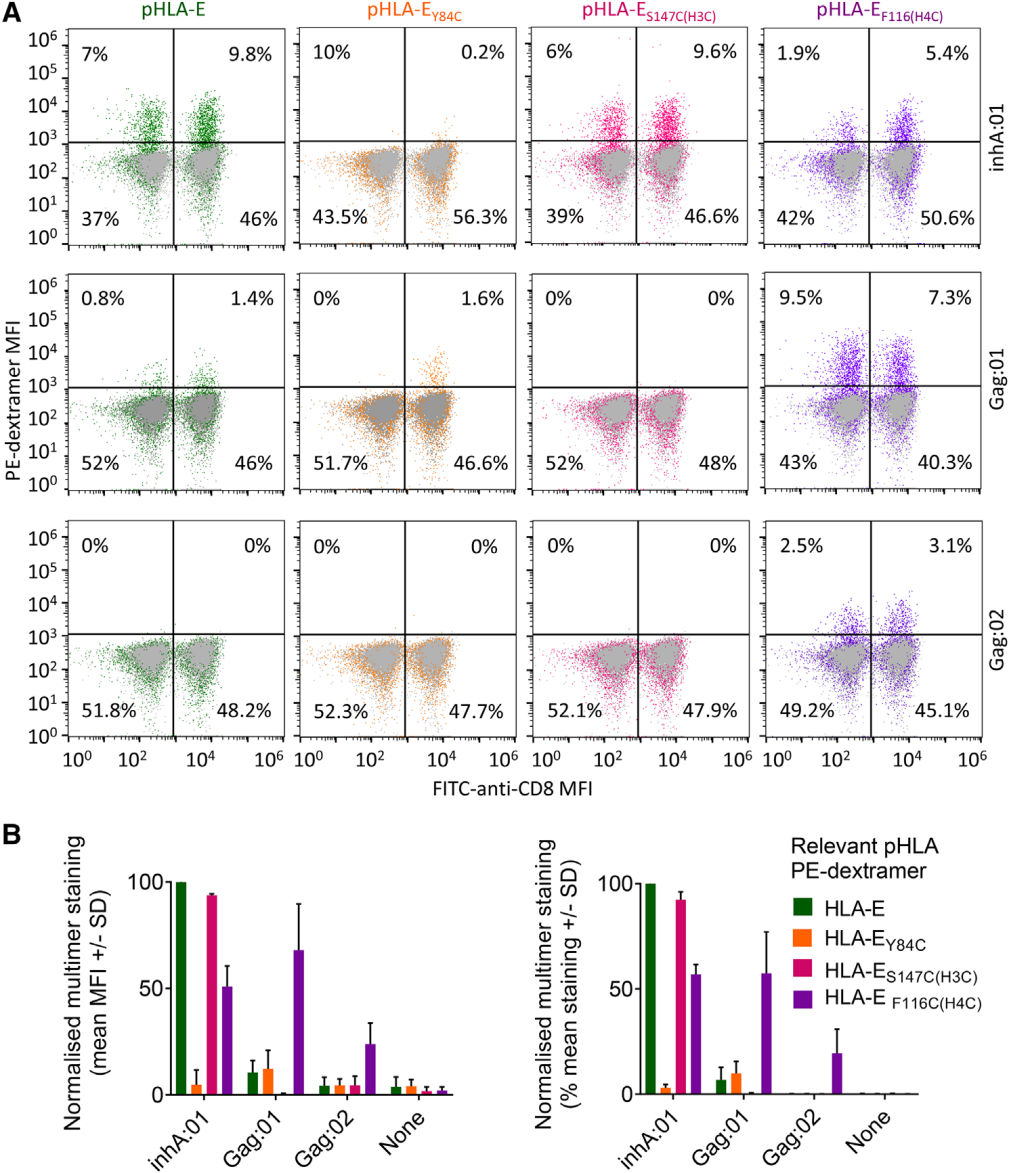
Identification of pHLA‐E‐specific T cells using WT, and non‐natural amino acid stabilized, pHLA‐E complex multimers. (A) Healthy PBMCs, lentivirally transduced to express WT TCRs recognizing HLA‐E‐inhA (inhA:01 TCR) or HLA‐E‐Gag6V (Gag6V:01, or Gag6V:02 TCRs) were stained with PE‐dextramers assembled with WT HLA‐E, HLA‐E_Y84C_, HLA‐E_S147C(H3C)_peptide, or HLA‐E_F116C(H4C)_peptide in complex with either inhA_53‐61_ or Gag6V_276‐284_. Flow cytometry was used to detect the % of dextramer positive CD8^+^, or CD8^‐^ T cells. Dot plots from one donor is shown as representative of n = 4 and n = 2 healthy PBMC donors for HLA‐E‐inhA or HLA‐E‐Gag6V‐specific TCRs, respectively. (B) Combined data from all donors as described in (A) normalized to PBMCs transduced with the inhA:01 TCR stained with WT HLA‐E‐inhA dextramer. Dextramer positive CD8^+^ T‐cell staining represented as MFI (Left) and percentage stained with each dextramer (Right) is shown.

Fluorescent multimers, such as tetramers, pentamers, and dextramers, are useful tools for identifying, isolating, and characterizing pHLA‐specific T cells. To test the utility of NNAA stabilized pHLA‐E in multimer assays, dextramers [29] were assembled and used to stain healthy PBMCs transduced to express inhA:01, Gag:01, or Gag:02 TCRs. Dextramer staining of the CD8^+^ and CD8^‐^ T‐cell populations was assessed by MFI and percentage staining (Fig. 7 and Supporting information Fig S1B). For these experiments, all results were normalized to the inhA:01 transduced T cells stained with the relatively stable WT HLA‐E‐inhA dextramer. No staining of inhA:01 TCR expressing cells was seen for dextramers constructed using HLA‐E_Y84C_‐inhA (Fig. 7). However, inhA:01 staining was observed when using the HLA‐E_F116C(H4C)_inhA or HLA‐E_S147C(H3C)_inhA dextramers, in line with the biochemical data. Conversely, HLA‐E‐Gag6V dextramers using WT HLA‐E resulted in minimal staining of T cells expressing the Gag:01 or Gag:02 TCRs (Fig. 7). Similarly, the pHLA‐E_Y84C_‐Gag6V or pHLA‐E_S147C(H3C)_Gag6V complexes yielded only marginal improvements over background. In contrast, the use of the HLA‐E_F116C(H4C)_Gag6V dextramer substantially improved staining for both Gag:01 (mean 57.4% staining) and Gag:02 TCRs (mean 19.46% staining). No nonspecific staining of HLA‐E‐Gag6V‐specific TCRs was observed with inhA‐based dextramers or vice versa (data not shown), or of nontransduced PBMCs (Fig. 7). These results indicate that NNAA stabilized dextramers can be used to isolate and characterize pHLA‐E‐specific T cells, although more work will be required to investigate how these reagents can be used to isolate pHLA‐E‐specific T cells from patient blood.

## Discussion

HLA‐E has been shown to present peptides in response to cellular stress [30], and has more recently been shown to play a role in regulating immunity in the cancer setting [31]. HLA‐E also has a less established role in presenting peptides from pathogenic origin during T‐cell antigen surveillance [10, 11, 32], with emerging evidence building toward the physiological relevance of this pathway [11, 13].

Here, we provide new insights into the nature of peptide presentation by HLA‐E, including the characterization of the structures of TCRs in complex with biologically characterized pathogen‐derived peptides from Mtb (inhA_53‐61_) and HIV (Gag6V_276‐284_) presented by HLA‐E. We found that the binding modes of these two TCRs were divergent, with the inhA:01 TCR recognizing the HLA‐E‐inhA in a very similar manner to the previously published KK50.4‐HLA‐E‐UL40 complex [9], making focused interactions with a single peptide residue (K5). In contrast, the Gag6V:02 TCR made a much broader set of interactions with the peptide, partly driven by the unique noncanonical peptide conformation compared to other pHLA‐E complexes [6, 9, 10]. These findings add to the recently reported structure of the GF4 TCR in complex with HLA‐E‐UL40 (in this report the authors solved the co‐complex structure of the GF4 TCR with UL40 and UL40 with the I8V mutation) demonstrating that the GF4 TCR utilized a unique binding mode to engage HLA‐E‐UL40 compared to the KK50.4 TCR [24]. Combined, these findings suggest that TCRs that recognize pHLA‐E can use divergent binding modes, mirroring classical TCR‐pHLA‐I complexes [18, 25, 26] in which the canonical diagonal docking orientation enables a variety of interactions with both the peptide and HLA surface. This divergent binding mode is in contrast to the more conserved binding mode observed for the recognition of some other nonpolymorphic HLA‐I‐like molecules including iNKT TCR‐CD1d [33] and MAIT TCR‐MR1 [34] complexes.

Although the nonpolymorphic nature of HLA‐E makes it an attractive therapeutic target, evidence suggests that some pathogen‐derived peptides might form unstable pHLA‐E complexes [4, 5, 10]. Here, we provide the first comprehensive assessment of pHLA‐E stability across a range of well‐characterized and putative pathogenic epitopes and HLA class Ia leader peptides. We show that pHLA‐E complexes can have a wide range of stabilities, with some previously described epitopes characterized by a *t*_1/2_ of as little as 0.16 h at 25°C. These findings have interesting implications for the biological role of HLA‐E, suggesting that HLA‐E turnover at the cell surface has the potential to be extremely rapid, which could enable a virtual real‐time representation of cellular status for NK and T‐cell interrogation. However, the instability of some HLA‐E peptides poses challenges for understanding the nature of NK or T‐cell recognition of pHLA‐E ligands, and for the development of pHLA‐E as a druggable target.

We used our co‐complex crystal structures as a foundation to assess multiple stabilization strategies including previously published examples for HLA class Ia molecules utilizing single (Y84C) or double (Y84C/A139C) heavy chain mutations [19, 20]. The Y84C/A139C mutation, which acts only to stabilize the peptide binding groove, was unable to offer significant improvements to pHLA‐E complex stability, rendering this stabilization mechanism incompatible with HLA‐E. In contrast, the Y84C mutation, coupled with the introduction of a cysteine residue on the peptide (+GCG extension) to create a cysteine trap, was successful at stabilizing pHLA‐E complexes and, in line with recent evidence [13], could be used to isolate antigen‐specific TCRs. However, structural analysis demonstrated the introduction of conformational changes could alter TCR binding affinity and TCR selection, akin to what has been observed for heteroclitic peptides in HLA class Ia molecules [28, 35, 36, 37]. We speculate that the artificial addition of residues to the C‐terminus of the peptide, and the nonoptimal distance between introduced cysteine pairs in the HLA and peptide, mediates these changes to the epitope. Thus, the use of such stabilized reagents for the generation of pHLA‐E multimers could lead to the isolation of TCRs that recognize the modified form, but not the native form, of the epitope. This is particularly important because disease‐specific pHLA‐E restricted T cells could be very rare in the periphery, so the generation of high‐quality reagents that represent the native epitope is essential.

Next, we turned to NNAAs to fine tune the peptide cysteine trap approach. We identified multiple modifications that could stabilize pHLA‐E while maintaining native‐like TCR binding affinity and geometry. For the HLA‐E‐inhA‐ and HLA‐E‐UL40‐specific TCRs, the HLA‐E_S147C(H3C)_peptide mutation maintained native binding affinity and enabled the selection of antigen‐specific TCRs from libraries. However, although structural analysis of the inhA:01‐HLA‐E_S147C(H3C)_inhA complex demonstrated a native‐like binding mode, we were unable to detect the introduced disulfide in the crystal structure or by MS analysis. Furthermore, we found that the HLA‐E_S147C(H3C)_peptide mutation completely abrogated binding by the HLA‐E‐Gag6V‐specific TCRs, possibly due to the C‐terminally shifted peptide bulge being adjacent to this modification, and differences in the recognition mode of this epitope (e.g., the broader peptide recognition might render HLA‐E‐Gag6V‐specific TCRs more sensitive to modifications that alter peptide dynamics compared to the HLA‐E‐inhA TCRs that were more “hot‐spot” driven). In contrast, the HLA‐E_F116C(H4C)_peptide mutation worked relatively well for HLA‐E‐inhA and HLA‐E‐UL40 TCRs, and was preferred for HLA‐E‐Gag6V TCRs, in terms of maintaining binding affinity, and for the selection of antigen‐specific TCRs from phage libraries. Structural analysis of TCRs in complex with HLA‐E_F116C(H4C)_inhA_/_UL40_/_Gag6V demonstrated a virtually identical protein‐protein interface for all three antigens, and the artificial disulfide formation was confirmed by both the crystal structures and MS analysis.

Finally, we demonstrated that NNAA engineered pHLA‐E complexes could be used to generate stable pHLA‐E multimers. Consistent with our other findings, multimers composed of the modified HLA‐E_S147C(H3C)_peptide complexes were the most successful for the isolation of HLA‐E‐inhA, but not HLA‐E‐Gag6V, specific T cells. In contrast, the HLA‐E_F116C(H4C)_peptide complexes could be used to identify antigen‐specific T cells against both epitopes from a mixed PBMC population. These findings demonstrate proof‐of‐concept for the use of the NNAAs to stabilize inherently unstable disease‐relevant pHLA‐E complexes. Future work will need to determine whether this approach can be used to successfully isolate patient‐derived T cells.

In summary, our data provide new insight into the nature of peptide presentation by HLA‐E including the characterization of co‐complex structures between TCRs and pathogen‐derived HLA‐E complexes. This structural analysis enabled characterization of the molecular impact of stabilizing modifications in the pHLA‐E complex and the development of alternative strategies using NNAAs to stabilize pHLA‐E while maintaining a native TCR recognition mode. These approaches led to the generation of stable, conformationally relevant, pHLA‐E monomers, representing an attractive reagent for characterizing HLA‐E‐specific T cells during pathogen surveillance, and as a platform to develop new pHLA‐E directed pan‐population therapies.

## Materials and methods

### Production of soluble pHLA‐E complexes

HLA‐E heavy chain (without transmembrane domain and with or without a C‐terminal biotinylation tag, AviTagsequence) and β2m were expressed and refolded with the peptide of interest and subsequently purified, as previously described [38]. To biotinylate complexes prior to size exclusion chromatography, complexes were AviTag treated with biotin‐protein ligase (BirA) according to the manufacturer's instructions (Avidity BirA‐500 kit) [39].

### Assessment of pHLA‐E complex stability

The stability of all pHLA‐E complexes was assessed by SPR using a BIAcore T200 instrument. Purified biotinylated pHLA‐E monomers (HLA‐E*01:03 was used throughout) were immobilized onto a streptavidin‐coupled CM5 sensor chip. A total of 1 μM of soluble ILT2 was flowed over the chip at 10 μL/min for 60 s. ILT2 binding to pHLA‐E complexes was measured at regular intervals over 5 h and responses were normalized by subtracting the bulk buffer response of a control flow cell containing no pHLA. Binding *t*_1/2_ was calculated by plotting % activity against time using the Biacore T200 evaluation software version 3.0 and GraphPad Prism version 8.3.0.

### TCR binding affinity analysis

Purified TCR molecules were produced as previously described [40] and subjected to SPR analysis using either a BIAcore T200 (for weak affinity molecules) or a BIAcore 8K system (for intermediate to strong affinity molecules). Briefly, biotinylated cognate pHLA‐E was immobilized onto a streptavidin‐coupled CM5 sensor chip. Flow cell one was loaded with free biotin alone to act as a control surface. *K_D_* values were calculated assuming Langmuir binding and data were analyzed using a 1:1 binding model (GraphPad Prism v8.3.0 for steady‐state affinity analysis and Biacore Insight Evaluation version 2.0.15.12933 for single cycle kinetics analysis).

### pHLA Thermofluor assay

Thermal shift assay experiments were performed using a RT‐PCR instrument (Quantstudio 6, Applied Biosystems). SYPRO Orange protein gel stain (5000× stock solution) was diluted in PBS to yield a 75× working solution. In a 96‐well plate, 23 μL of pHLA at 0.25 mg/mL was added to 2 μL of SYPRO Orange working solution to a final volume of 25 μL, with each pHLA prepared in triplicate. Mixtures were heated from 10 to 95°C with a temperature increment of 1°C/min. Fluorescence was detected using the FAM filter set, with excitation and emission wavelength at 495 and 518 nm, respectively. Data analysis was performed using Protein Thermal Shift Software version 1.4 (Thermofisher Scientific) to determine *Tm*.

### Cell culture

K562 cells transduced with single chain HLA‐E*01:03‐β2m were routinely cultured in cRPMI‐10, consisting of RPMI‐1640 media (Gibco, Cat. No. 42401‐018) supplemented with 1% v/v penicillin/streptomycin, 2 mM l‐glutamine, 10% fetal bovine serum (Gibco, Cat. No. 10438‐026, pretested for assay performance) at 37°C/5% CO_2_. PBMCs were cultured in cRPMI‐10 supplemented with 50 U/mL IL‐2.

### Flow cytometry stability analysis of peptide pulsed cells

All flow cytometry experiments were performed according to MIFlowCyt guidelines [41] K562 cells transduced with single chain HLA‐E*01:03‐β2m were either left unpulsed or pulsed with 10 μg/mL peptide for 2 h at 37°C/5% CO_2_. Immediately following peptide pulsing, cells were washed once with wash buffer (PBS + 2 nM EDTA + 2% human AB serum [Sigma Aldrich, Cat. No. H3667]) and either left unstained or stained for 30 min at 4°C using anti‐human HLA‐E‐PE (3D12; BioLegend) or anti‐mouse IgG1κ‐PE (MOPC‐21; BD Pharmingen). Flow cytometry was performed using a Sony SH800S (Sony Biotechnology, software version 2.1.5.) loaded with a 100 μm sorting chip (Sony Biotechnology, Cat. No. LE‐C3210) and calibrated with automatic setup beads (Sony Biotechnology, Cat. No. LE‐B3001). Samples were washed twice before a minimum of 20,000 gated singlets were analyzed per sample (gating as per Supporting information Fig. S1). Cytometer files were exported (FCS files and full instrument settings available on request) and analyzed with FlowJo software (FlowJo LLC version 10.7.1).

### Protein crystallization

The TCR‐pHLA‐E complexes were prepared by mixing purified TCR and pHLA‐E at a molar ratio of 1:1.15 and concentrating to approximately 10 mg/mL. The crystallization trials were set up by dispensing 150 nL of protein solution plus 150 nL of reservoir solution in sitting‐drop vapor diffusion format in two‐well MRC Crystallization plates using a Gryphon robot (Art Robbins). The plates were maintained at 20°C in a Rock Imager 1000 (Formulatrix) storage system. Diffraction quality crystals were obtained in the following conditions: inhA:01‐HLA‐E‐inhA and inhA:01‐HLA‐E_Y84C_‐inhA (0.1 M TRIS pH 8.5, 15 % glucose and 25 % w/v PEG 4000); inhA:01‐HLA‐E_S147C(H3C)_inhA (0.1 M Sodium chloride, 0.1 M Bis‐Tris pH 6.5, and 1.5 M Ammonium sulfate); inhA:01‐HLA‐E_F116C(H4C)_inhA (0.1 M Tris pH 8.5, 15% glucose, and 20% w/v PEG 4000); KK50.4‐HLA‐E_F116C(H4C)_UL40 (55 mM MOPS pH 6.5 and 12% w/v PEG 8000); Gag6V:02‐HLA‐E‐Gag6V (93 mM MMT pH 9.0 and 23.2% w/v PEG 1500); and Gag6V:02‐HLA‐E_F116C(H4C)_Gag6V (89 mM MMT pH 9.0 and 22.3% w/v PEG1500).

### X‐ray data collection and structure determination

Crystals were cryoprotected using reservoir solution supplemented with 30% v/v ethylene glycol and then flash cooled in liquid nitrogen. X‐ray diffraction data were collected at the Diamond Light Source (Oxfordshire, UK) beamlines I04, I04‐1, and I03. Diffraction images were indexed, integrated, scaled, and merged using XDS and XSCALE [42, 43] or dials and dials.scale [44, 45] through the xia2 automated data‐processing suite [46]. Structures were solved by molecular replacement using Phaser [47], the search models used were PDB 5W1W (chains A and B) for HLA‐E, PDB 5EU6 (chains D and E) for the inhA:01 and Gag:02 TCRs, and 2ESV (chains D and E) for the KK50.4 TCR. Models were built using iterative cycles of manual model building in COOT [48] and refinement using Refmac [49] in the CCP4 suite [50]. Additional model validation and assessment of the stereochemical properties of the models was performed using PDB_REDO [51, 52] and the PDB Validation Suite [53]. The data processing and refinement statistics are listed in Supporting information Table S3. The structural figures were prepared using PyMOL (Schrödinger). All structural superpositions were performed using SSM [54].

### Mass spectrometry

To analyze covalent bond formation between the HLA‐E heavy chains and peptide, biotinylated WT HLA‐E‐inhA, HLA‐E‐Gag6V, HLA‐E_Y84C_‐inhA, HLA‐E_Y84C_‐Gag6V, HLA‐E_S147C(H3C)_inhA, HLA‐E_S147C(H3C)_Gag6V, HLA‐E_F116C(H4C)_inhA, HLA‐E_F116C(H4C)_Gag6V, untagged WT HLA‐E‐Gag6V, HLA‐E‐UL40, HLA‐E_F116C(H4C)_Gag6V, and HLA‐E_F116C(H4C)_UL40 were analyzed using an AB SCIEX Triple TOF 6600 ESI mass spectrometer in conjunction with a RS3000 series HPLC system. The HPLC system was essentially used as a desalting step, where up to 5 μg of total protein was loaded onto a MAbPac RP 4 μm 2.1 × 50 mm rpHPLC column and eluted in a steep gradient of acetonitrile in a background of water‐formic acid at flow rate of 250 μL a minute. The Triple TOF 6600 mass‐spectrometer, configured for intact protein work, was set up with the TurboSpray Ion source to accommodate the high flow rates from the HPLC and operated at a spray voltage of 5500 V. In addition, the instrument was configured in TOF‐MS mode only, positive polarity, using a full scan range of 400–2500 and an accumulation time of approximately 1 s. The complex spectra obtained were subject to deconvolution using the AB‐SCIEX software PeakView (v2.1) and the add‐in package BioTool Kit.

### TCR discovery using phage panning

Naïve TCR phage display libraries were used for TCR discovery, with phage display carried out as previously described [55]. The inhA, UL40, or Gag6V TCRs were generated through panning using WT HLA‐E, HLA‐E_Y84C_, HLA‐E_S147C(H3C)_peptide, and HLA‐E_F116C(H4C)_peptide for selection with their cognate peptides.

### ELISA

Productive clones from panning outputs were grown in 2×YT supplemented with ampicillin and glucose in 96‐well plates (AB‐1127, Thermo Fisher Scientific) for 2 h prior to infection with KM13 helper phage at a MOI of 10 for 30 min at 37°C. Plates were centrifuged and the cell pellets resuspended in 2× YT supplemented with ampicillin and kanamycin, and cultures allowed to grow overnight at 26°C. Phage was precipitated on ice using PEG/NaCL, and the precipitated phage resuspended in PBS for use in the ELISA. Phage was blocked at a 1:1 ratio in 6% milk for 1 h, while pHLA‐E complexes at 5 μg/mL were used to coat Maxisorp Immuno ELISA plates (Thermo Fisher Scientific, 439454) previously incubated overnight at 4°C with streptavidin (5 μg/mL). WT HLA‐E, HLA‐E_Y84C_, HLA‐E_S147C(H3C)_peptide, and HLA‐E_F116C(H4C)_peptide in complex with inhA, UL40, or Gag6V peptides, as well as a mixture of pHLA‐E leader sequences to identify cross‐reactive TCRs, were used in the ELISA. pHLA‐E‐coated ELISA plates were washed three times prior to blocking with 3% milk for 30 min, before the addition of the blocked phage to the ELISA plates for 30 min at room temperature. Plates were washed four times, and detection performed by adding αM13‐HRP antibody diluted 1:5000 in 0.5% BSA (Sigma, A3059) for 30 min, followed by the detection substrate (TMB Microwell Peroxidase Substrate System; KPL Labs 50‐76‐00). Colorimetric change was read at OD_650_ using a plate reader.

### Lentiviral transduction of PBMCs with pHLA‐E‐specific TCRs

Plasmids encoding TCRs specific for HLA‐E‐Gag6V or HLA‐E‐inhA were designed and cloned for use in lentiviral transductions. Human codon optimized sequences of inhA:01, Gag:01, and Gag:02 TCRs were synthesized by GeneArt (Thermo Fisher Scientific, , Boston, MA, USA) and cloned into the pELNS transfer vector using 5’ NheI and 3’ SalI restriction sites. To generate lentivirus, plasmids were transfected into HEK293T cells using Turbofect transfection reagent (Thermo Fisher Scientific). Lentiviral particles were harvested and used to transduce isolated PBMCs. One day prior to lentiviral transduction, heparanized whole blood was obtained from four healthy volunteers by venipuncture. The Oxford A REC approved protocol 13/SC/0226 was used to obtain written consent for all blood donations and was fully approved by the National Research Ethics Committee South Central. PBMCs were immediately isolated from 50 mL whole blood per donor by density centrifugation using Ficoll–Hypaque. PBMCs were counted on a Cellometer Auto 2000 (Nexcelom Bioscience) using AO/PI to assess cell yield (median yield 2.3 × 10^6^/mL in 10 mL) and cell viability (median 97.55%). PBMCs were cultured at 37°C/5% CO_2_ in six‐well plates with 1 mL × 1 × 10^6^ viable cells per well in cRPMI‐10 supplemented with 50 U/mL IL‐2 and human T‐Activator CD3/CD28 microbeads (three beads/cell; Life Technologies Cat. No. 11131D). The following day, lentivirus was added directly to each well except for a nontransduced control well for each PBMC donor. Microbeads were removed by magnetic separation 5 days post‐transduction and fresh cRPMI‐10 supplemented with 50 U/mL IL‐2 was added to each well every 2–3 days, but not within 72 h of dextramer staining.

### pHLA‐E dextramer staining and FACs analysis of TCR‐transduced PBMCs

TCR‐transduced and nontransduced PBMCs were transferred to v‐bottom 96‐well plates (50,000–100,000 cells/sample) and washed with buffer (phenol red‐free HBSS + 1% human AB serum (Sigma Aldrich, Cat. No. H3667) + 2 mM EDTA + 10 mM HEPES). Cell pellets were resuspended in 30 μL/well buffer + 50 nM dasatanib (Selleckchem Cat. No. S1021) and incubated for 30 min at 37°C/5% CO_2_. Klickmer/PE dextramer backbone (Cat. No. DX01‐PE; Immudex, Denmark) was assembled with WT and trapped versions of biotinylated pHLA‐E to a final concentration of 32 nM as per the manufacturer's instructions. PBMCs were immediately stained with PE‐dextramer by adding 0.5 μL assembled dextramer directly to the dasatinib‐treated cells and incubating at 4°C for 30 min. PBMCs were washed twice in cold buffer and then stained with anti‐CD8‐BB515 (BD Biosciences Cat. No. 564526) and Molecular Probes live/dead Fixable Violet (Invitrogen Cat. No. L‐34955) for 20 min at 4°C. During staining, a Sony SH800S (Sony Biotechnology, software version 2.1.5) flow cytometer was loaded with a 100 μm sorting chip (Sony Biotechnology, Cat. No. LE‐C3210) and calibrated with automatic setup beads (Sony Biotechnology, Cat. No. LE‐B3001). Automatic compensation was carried out using unstained (negative control) and single‐stained compensation beads (BD Biosciences, Cat. No. 552843) to generate a spillover matrix for compensation (full instrument settings available on upon request). PBMCs were washed twice with cold buffer and kept at 4°C before a minimum of 20,000 total events were analyzed per sample. Gating was performed as shown in Supporting information Fig. S1. Cytometer files were exported (FCS files available upon request) and analyzed with FlowJo software (FlowJo LLC version 10.7.1) and values normalized to PBMCs transduced with the inhA:01 TCR stained with WT HLA‐E‐inhA‐PE‐dextramer.

## Funding

This study was funded in its entirety by Immunocore Ltd.

## Author contributions

CB, VAD, RLP, MM‐U, NCM, VS, MC, TF‐L, GP, RP, VK, TG, MD, AV, DH, THB, AK, RAR, DKC, and SL conducted experiments and/or contributed toward experimental design. CB, DKC, and SL wrote the article. DKC and SL conceived and/or directed the project. All authors critiqued the manuscript.

## Conflict of interest

All authors are, or were, employees of Immunocore Ltd.

### Peer review

The peer review history for this article is available at https://publons.com/publon/10.1002/eji.202149745.

**Abbreviations**
HBV: hepatitis B virus
Mtb: *Mycobacterium tuberculosis*
NNAA: non‐natural amino acid
SPR: surface plasmon resonance
*Tm*
: thermal melting point

## Supporting information

Supporting information

## Data Availability

All structures were deposited in the protein data bank under the following accession codes: inhA:01‐HLA‐E‐inhA PDB accession code: 6ZKW inhA:01‐HLA‐E_Y84C_‐inhA PDB accession code: 6ZKX inhA:01‐HLA‐E_S147C(H3C)_inhA PDB accession code: 6ZKY inhA:01‐HLA‐E_F116C(H4C)_inhA PDB accession code: 6ZKZ UL40:01‐HLA‐E_F116C(H4C)_UL40 PDB accession code: 7NDT Gag6V:02‐HLA‐E‐Gag6V PDB accession code: 7NDQ Gag6V:02‐HLA‐E_F116C(H4C)_Gag6V PDB accession code: 7NDU
