# Supplementary material for: Structure‐guided stabilization of pathogen‐derived peptide‐HLA‐E complexes using non‐natural amino acids conserves native TCR recognition
Source: Eur J Immunol. 2022 Feb 13;52(4):618–32. doi: 10.1002/eji.202149745 (PMC9306587; doi:10.1002/eji.202149745)
Supplement: Supplementary file 1 — Supporting information [file EJI-52-618-s001.pdf]

Peptide	Sequence present in HLA-E allele
VMAPRTLVL	**A2**, A23, A24, A25, A26, A43, A66, A68, A69
VMAPRTLIL	**Cw3**, Cw1, Cw4, Cw5, Cw6, Cw8, Cw12, Cw13, Cw15
VMAPRTLLL	**A1**, A3, A11, A29, A30, A31, A32, A33, A36, A74, Cw2, Cw14
VMAPRTLFL	**G**
IMAPRTLVL	**A34**
VMPPRTLLL	**A80**
VMAPRTVLL	**B7**, B8, B14, B38, B39, B42, B48, B67, B73, B81
VTAPRTLLL	**B13**, B18, B27, B37, B40:05, B44, B47, B54, B55, B56, B59, B82
VTAPRTVLL	**B15**, B35, B40, B41, B45,B46, B49, B50, B51, B52, B53, B57,B58, B78
VMAPRALLL	**Cw7**, Cw17, Cw18
VMAPQALLL	**Cw16**

Supplementary Table S1: HLA-derived leader sequences.
11 peptide sequences cover all HLA-A, -B, -C, and -G alleles. Letters in bold represent the name of leader sequences used in Figures and Tables throughout.

Peptide Origin	Peptide Name	Peptide Sequence	$t_{1/2}$ (mins)	$t_{1/2}$ (hours)	Average $_{Tm}$ (°C)
Mtb	$InhA_{53-61}$	RLPAKAPLL	231.4	3.86	48.23
Mtb	$mmpL8_{75-83}$	ILPSDAPVL	39.57	0.66	44
Mtb	$ppsB_{483-491}$	RMAATAQVL	13.39	0.22	40.54
Mtb	$rv1518_{240-248}$	VMATRRNVL	19.35	0.32	ND
Mtb	$rv2997_{470-478}$	RMPPLGHEL	18.44	0.31	ND
Mtb	$rv3428c_{226-234}$	ALPPRAFEL	9.34	0.16	43.65
CMV	CMV $UL40_{15-23}$	VMAPRTLIL	177.8	2.96	47.81
HIV Gag	$Gag6T_{276-284}$	RMYSPTSIL	11.39	0.19	ND
HIV Gag	$Gag6V_{276-284}$	RMYSPVSIL	9.56	0.16	ND
HCV Core	HCV $Core_{36-44}$	LLPRRGPRL	13.86	0.23	ND
HSP 60	$hsp60sp_{10-18}$	QMRPVSRVL	9.94	0.17	40.33
Leader A1	$LA1_{3-11}$	VMAPRTLLL	168	2.8	47.87
Leader A2	$LA2_{3-11}$	VMAPRTLVL	186.9	3.12	47.6
Leader A34	$LA34_{3-11}$	IMAPRTLVL	193.4	3.22	47.76
Leader A80	$LA80_{3-11}$	VMPPRTLLL	186.5	3.11	47.53
Leader B7	$LB7_{3-11}$	VMAPRTVLL	207.9	3.47	47.47
Leader B13	$LB13_{3-11}$	VTAPRTLLL	11.42	0.19	43.73
Leader B15	$LB15_{3-11}$	VTAPRTVLL	13.62	0.23	41.93
Leader Cw7	$LCW7_{3-11}$	VMAPRALLL	76.17	1.27	46.53
Leader Cw16	$LCW16_{3-11}$	VMAPQALLL	18.51	0.31	43.47
Leader G	LG_{3-11}	VMAPRTLFL	141	2.35	47.33

Supplementary Table S2. pHLA-E stability as assessed by BIACore and Thermofluor.
Mtb = *Mycobacterium tuberculosis*
HCV = Hepatitis C virus
HSP = Heat shock protein
HIV = Human immunodeficiency virus
CMV = Cytomegalovirus
NB = Not determined

	inhA:01 TCR complexes				Gag:02 TCR complexes		KK50.4 TCR complex
Molecule	inhA:01-HLA-E-inhA	inhA:01-HLA-E_{Y84C}-inhA	inhA:01-HLA-$E_{S147C(H3C)}$ inhA	inhA:01-HLA-$E_{F116C(H4C)}$ inhA	Gag:02-HLA-E-Gag6V	Gag:02-HLA-$E_{F116C(H4C)}$ Gag6V	KK50.4-HLA-$E_{F116C(H4C)}$ UL40
PDB code	6ZKW	6ZKX	6ZKY	6ZKZ	7NDQ	7NDU	7NDT
Space group	$P\ 2_1\ 2_1\ 2_1$	$P\ 2_1\ 2_1\ 2_1$	*P* 21 21 21	$P\ 2_1\ 2_1\ 2_1$	$P\ 4_3\ 2_1\ 2$	$P\ 4_3\ 2_1\ 2$	$P\ 1\ 2_1\ 1$
Unit cell dimensions	a=71.14, b=108.67, c =119.94; α, β, γ = 90°	a=71.87, b=107.86, c=119.04; α, β, γ = 90°	a=72.04, b=108.87, c=119.45; α, β, γ = 90°	a=70.81, b=107.89, c=118.42; α, β, γ = 90°	a=89.39, b=89.39, c=293.30; α, β, γ = 90°	a=88.98, b=88.98, c=293.57; α, β, γ = 90°	a=117.61 b=76.51 c=130.34 α=90°, β=107.64°, γ=90°
X-ray source	DLS I04	DLS I04-1	DLS I03	DLS I04-1	DLS I04	DLS I04	DLS I04
Wavelength (Å)	0.9795	0.9159	0.9763	0.9119	0.97950	0.97950	0.97951
Resolution range (Å)	59.97 - 2.26 (2.30 - 2.26)[a]	79.93 - 2.17 (2.21 - 2.17)[a]	60.08 - 2.65 (2.70 - 2.65)[a]	70.81 – 2.30 (2.34 - 2.30)[a]	76.33-2.55 (2.59-2.55)[a]	61.25-2.90 (2.95-2.90)[a]	65.19-3.00 (3.05-3.00)[a]
Completeness (%)	99.73 (97.95)	99.31 (98.89)	99.75 (95.76)	99.92 (98.96)	100.0 (99.8)	99.9 (99.2)	99.9 (99.3)
Multiplicity	6.8 (4.3)	7.3 (6.4)	15.0 (14.1)	14.8 (14.8)	26.3 (27.4)	8.8 (8.3)	3.4 (3.3)
I/σ I	14.3 (1.1)	12.8 (1.2)	15.2 (1.0)	19.7 (2.7)	11.6 (0.3)	8.6 (1.0)	13.4 (0.4)
R_{merge}	0.069 (1.183)	0.102 (1.342)	0.141 (2.442)	0.081 (0.840)	0.207 (6.926)	0.230 (2.569)	0.101 (2.198)
R_{pim}	0.028 (0.616)	0.040 (0.579)	0.037 (0.657)	0.022 (0.223)	0.041 (1.343)	0.082 (0.933)	0.064 (1.425)
$CC_{1/2}$	0.999 (0.352)	0.999 (0.581)	0.999 (0.615)	0.999 (0.935)	1.000 (0.735)	0.998 (0.458)	0.995 (0.134)
Unique reflections	44167 (2150)	49326 (2415)	27789 (1288)	40998 (1989)	39941 (1932)	27166 (1306)	44666 (2218)
Refinement							
Rwork / Rfree (%)	24.3 / 28.7	20.9 / 24.4	22.4 / 27.3	22.4 / 26.7	23.3 / 28.6	24.3 / 30.3	22.2 / 27.1
RMS (bonds)	0.0020	0.0020	0.0017	0.0019	0.0082	0.0065	0.0036
RMS (angles)	1.195	1.175	1.167	1.170	1.773	1.539	1.298
Mean B-factor ($Å^2$)	61.85	45.68	79.64	55.41	95.24	69.221	70.61

Supplementary Table S3. X-ray data collection and refinements statistics.
[a]Values in the parentheses refer to the outer resolution shell

Peptide Name	Peptide Sequence	$t_{1/2}$ (hours)				
		HLA-E	HLA-E$_{Y84C}$	HLA-E$_{Y84C/A139C}$	HLA-E$_{S147C(H3C)}$	HLA-E$_{F116C(H4C)}$
inhA$_{53-61}$	RLPAKAPLL	3.86	23.76	4.75	16.01	>24
mmpL8$_{75-83}$	ILPSDAPVL	0.66	>24	1.06	8.61	NP
Rv1518$_{240-248}$	VMATRRNVL	0.32	>24	0.94	NP	NP
CMV UL40$_{15-23}$	VMAPRTLIL	2.96	>24	2.66	4.75	17.32
Gag6V$_{276-284}$	RMYSPVSIL	0.16	16.6	0.88	9.73	9.83
hsp60sp$_{10-18}$	QMRPVSRVL	0.17	NP	NP	9.37	NP
LA1$_{3-11}$	VMAPRTLLL	2.8	NP	NP	16.74	NP
LA2$_{3-11}$	VMAPRTLVL	3.12	>24	2.51	4.87	NP
LA80$_{3-11}$	VMPPRTLLL	3.11	NP	NP	9.84	NP
LB13$_{3-11}$	VTAPRTLLL	0.19	NP	NP	18.22	NP
LCW16$_{3-11}$	VMAPQALLL	0.31	NP	NP	>24	NP
LCW7$_{3-11}$	VMAPRALLL	1.27	NP	NP	16.08	NP
LG$_{3-11}$	VMAPRTLFL	2.35	NP	NP	23.96	NP

Supplementary Table S4. pHLA-E stability with engineered cysteine traps as assessed by BIACore.
NP = not performed

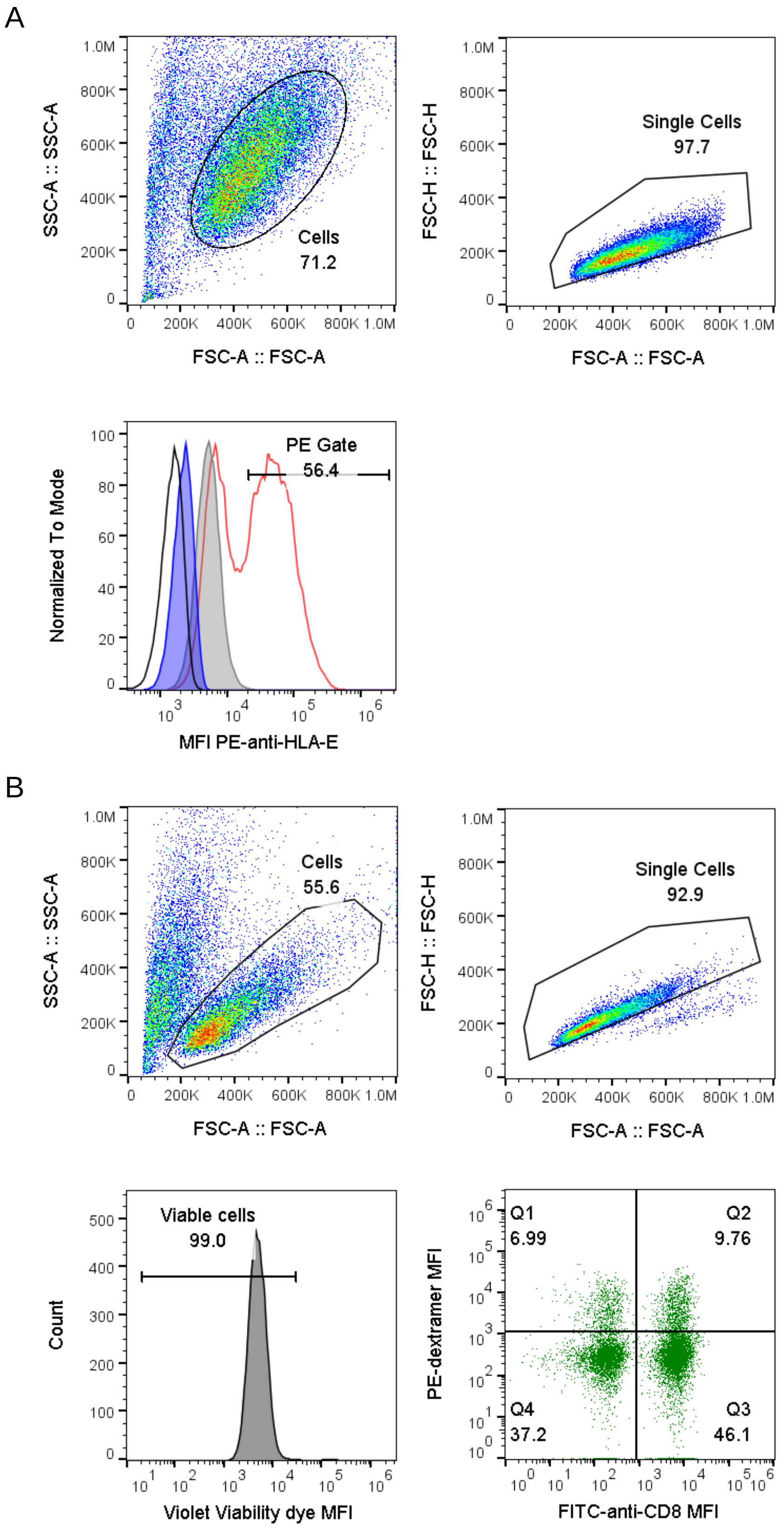

Supplementary Figure 1. Flow cytometry gating strategies to assess (**A**) cell surface HLA-E levels and (**B**) PE-dextramer staining. (**A**) K562 cells transduced with single chain HLA-E*01:03-β2m were analysed by gating on single cells then assessing the PE channel. Control samples include unstained cells (black line), cells stained with anti-mouse IgG1κ-PE isotype control (blue shading) or cells stained with anti-HLA-E-PE, either unpulsed (grey shading) or after pulsing with peptide such as positive control peptide inhA$_{53\text{-}61}$ (red line). The PE gate shown is represented as a measure of stabilisation of cell surface HLA-E levels after peptide pulsing with peptides of interest (Figure 1B). (**B**) The flow cytometry gating strategy used to analyze the singlet, viable cell population for FITC-anti-CD8 staining and PE-dextramer median fluorescence intensity and percentage staining (Figure 7). This example shows gating of PBMC transduced with the inhA:01 TCR stained with wildtype HLA-E-inhA dextramer.

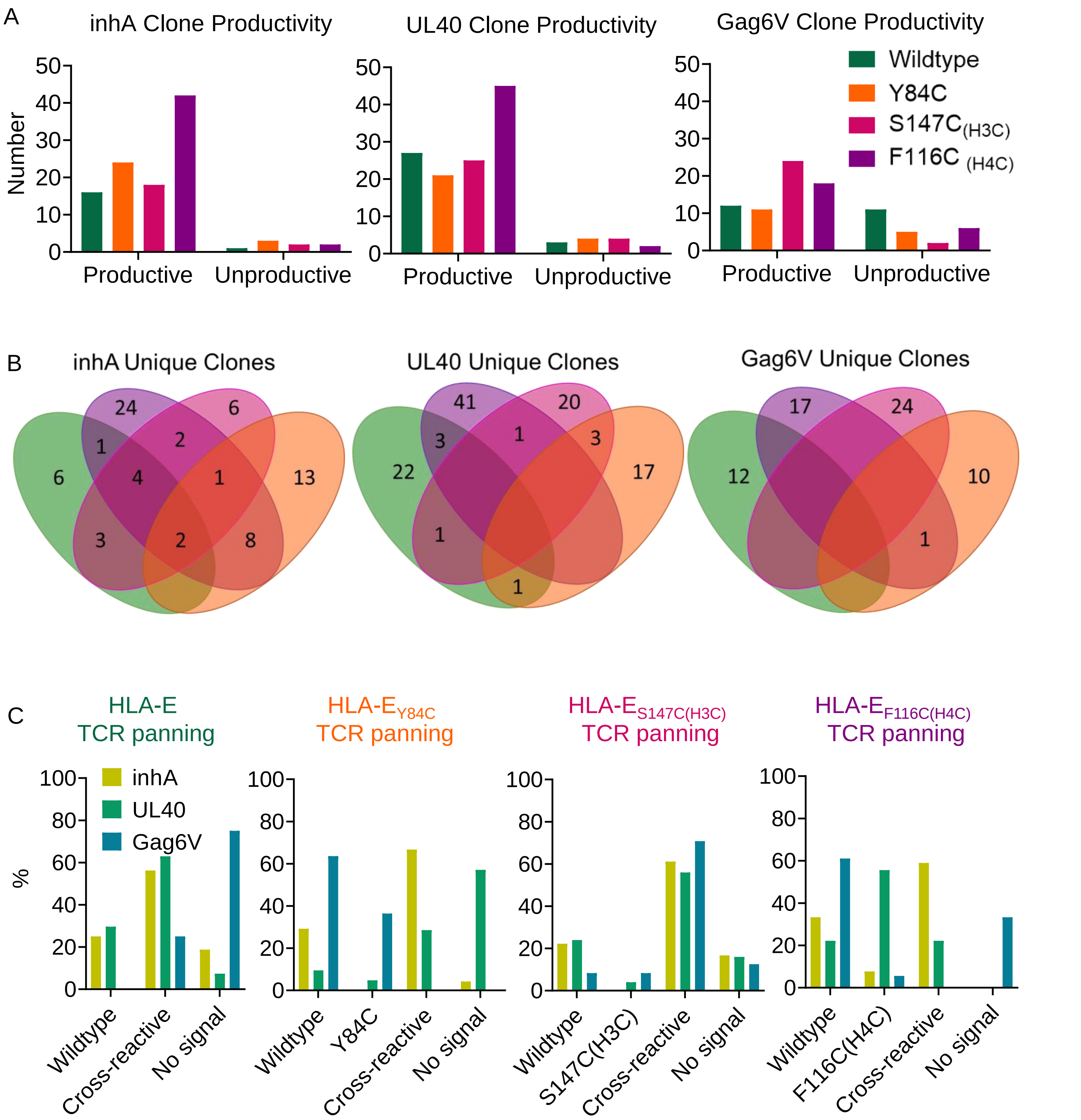

Supplementary Figure S2. TCR isolation from phage libraries by bio-panning using wildtype or stabilized pHLA-E as target.

(**A**) Number of unique productive TCR clones isolated from bio-panning against inhA, UL40 or Gag6V (as determined by the lack of stop codons present in the TCR or the absence of frame shifts) using wildtype (unmodified) pHLA-E (shown in green), pHLA-E_{Y84C} (orange), pHLA-$E_{S147C(H3C)}$peptide (pink), or pHLA-$E_{F116C(H4C)}$peptide (purple). (**B**) Venn diagrams of the unique clones identified through phage bio-panning using wildtype (unmodified) pHLA-E (shown in green), pHLA-E_{Y84C} (orange), pHLA-$E_{S147C(H3C)}$peptide (pink), or pHLA-$E_{F116C(H4C)}$peptide (purple). Clones common to multiple outputs are shown in the overlaid areas. (**C**) Data corresponding to bio-panning outputs from wildtype (unmodified) pHLA-E, pHLA-E_{Y84C}, pHLA-$E_{S147C(H3C)}$peptide and pHLA-$E_{F116C(H4C)}$peptide. Percentage of TCRs panned with each HLA-E modification against HLA-E-inhA (yellow), HLA-E-UL40 (green), HLA-E-Gag6V (blue) reactive against the wildtype HLA-E complex, cross-reactive with a mixed panel HLA-E leader peptides, or no signal in ELISA testing. Wildtype = TCRs that bind to the wildtype complex but not the cross-reactivity panel (TCRs might also bind to one or more of the HLA-E stabilized mutants). Cross-reactive = TCRs that bind to a mixture of leader peptides presented by HLA-E. Y84C/S147C(H3C)/F116C(H4C) = TCRs that bind to the stabilized pHLA-E, but not the wildtype or cross-reactivity panel.

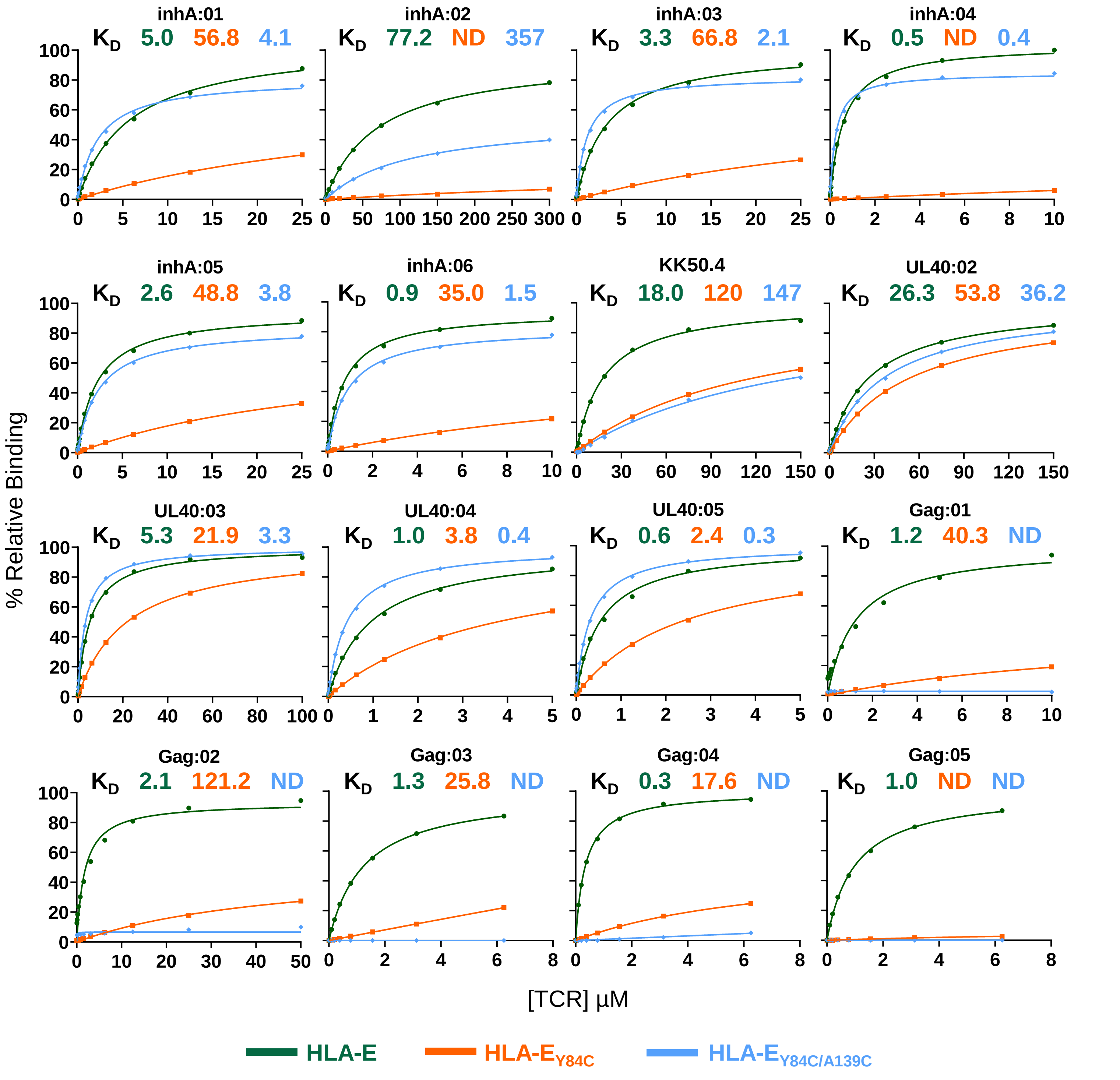

Supplementary Figure S3. TCR binding of conventionally trapped pHLA-E complexes.
TCR binding affinity, as assessed by surface plasmon resonance, depicting the equilibrium dissociation constant (K_D) for 6 TCRs specific to HLA-E-inhA, 5 TCRs specific to HLA-E-UL40 and 5 TCRs specific to HLA-E-Gag6V. All K_D values are shown in µM. Wildtype HLA-E is colored green, HLA-E_{Y84C} is colored orange and HLA-$E_{Y84C/A139C}$ is colored blue. Data representative of at least two independent experiments. ND = K_D could not be determined.

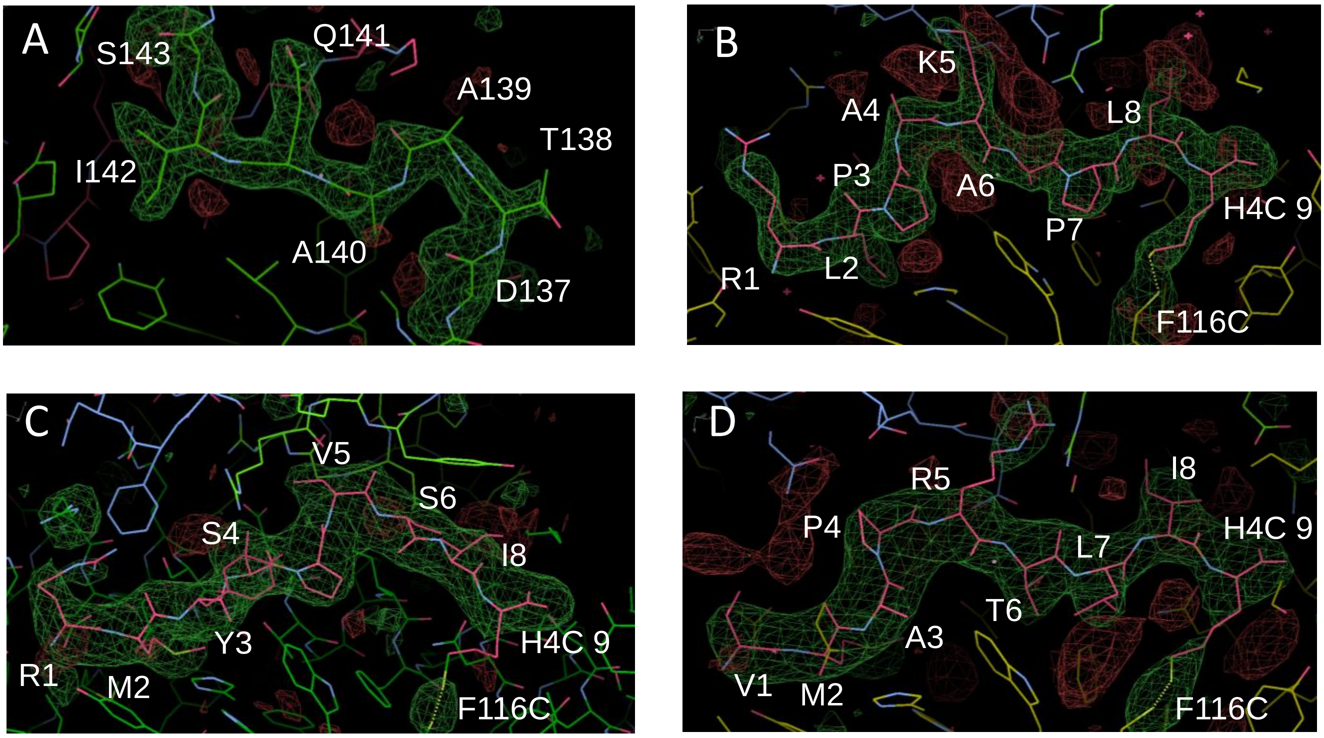

Supplementary Figure S4. Fo-Fc electron density omit maps.

Fo-Fc electron density difference maps (green mesh positive density red mesh negative density) contoured at +/- 3σ. (**A**) inhA:01-HLA-E$_{Y84C}$-inhA complex, showing re-arrangement of HLA-E residues D137-S143; map calculated with those residues omitted from the model. (**B-D**) maps calculated omitting the peptide and HLA-E residue F116C from the model. (**B**) inhA:01-HLA-E$_{F116C(H4C)}$inhA complex displaying continuous positive density for the peptide and the disulphide bond between H4C and HLA-E residue F116C. (**C**) Gag:02-HLA-E$_{F116C(H4C)}$-Gag6V complex displaying dis-continuous positive density for the disulphide bond between H4C and HLA-E residue F116C. (**D**) KK50.4-HLA-E$_{F116C(H4C)}$UL40 complex displaying continuous positive density for the peptide and the disulphide bond between H4C and HLA-E residue F116C.

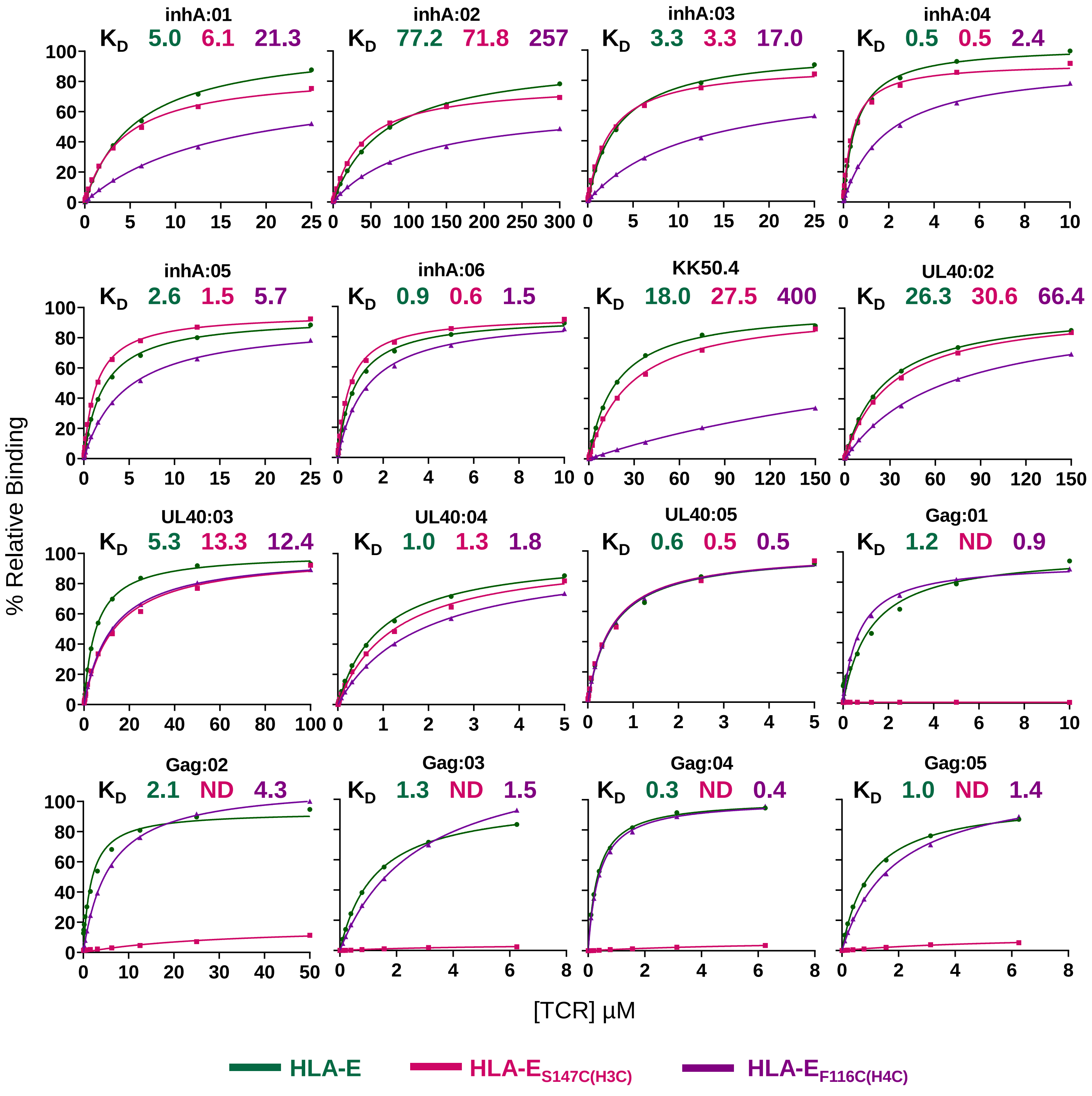

Supplementary Figure S5. TCR binding of non-natural amino acid trapped pHLA-E complexes. TCR binding affinity, as assessed by surface plasmon resonance, depicting the equilibrium dissociation constant (K_D) for 6 TCRs specific to HLA-E-inhA, 5 TCRs specific to HLA-E-UL40 and 5 TCRs specific to HLA-E-Gag6V. All K_D values are shown in µM. Wildtype HLA-E is colored green, HLA-$E_{S147C(H3C)}$peptide is colored pink and HLA-$E_{YF116(H4C)}$peptide is colored purple. Data representative of at least two independent experiments. ND = K_D could not be determined.

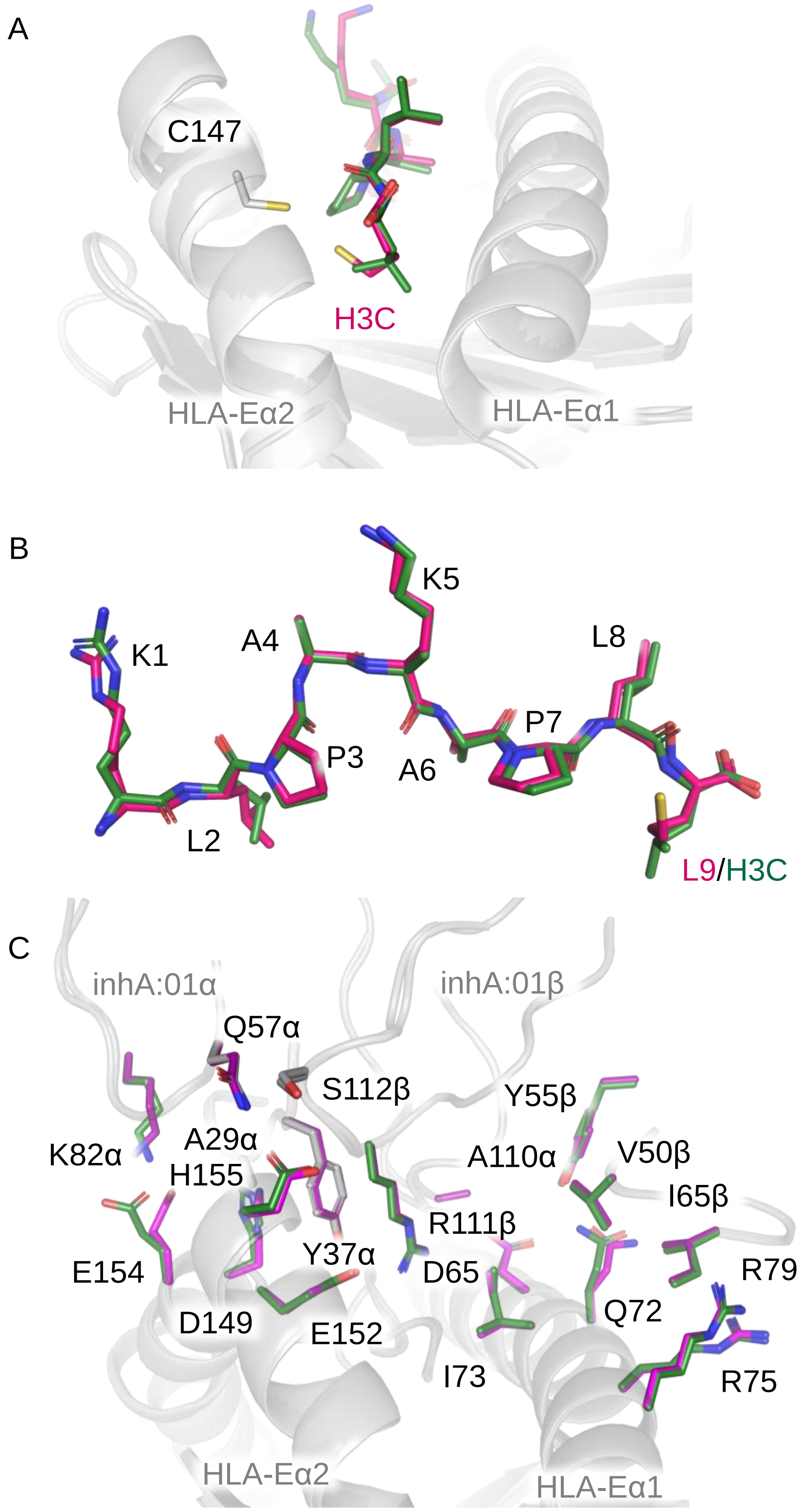

Supplementary Figure S6. Structural comparison of the inhA:01 TCR in complex with pHLA-E-inhA and HLA-E$_{S147C(H3C)}$inhA.

(**A**) Superposition of the wildtype HLA-E-inhA (HLA-E shown in gray cartoon, inhA shown in green sticks) and HLA-E$_{S147C(H3C)}$inhA (HLA-E$_{S147C}$ shown in gray cartoon, H3C inhA shown in pink sticks). The non-natural disulfide between C147 and inhA$_{53\text{-}61}$ is shown in yellow sticks. (**B**) Superposition of the wildtype HLA-E-inhA (green sticks), and HLA-E$_{S147C(H3C)}$inhA (pink sticks) structures. (**C**) Superposition of the inhA:01 TCR (gray cartoon) in complex with wildtype HLA-E-inhA (gray cartoon), or HLA-E$_{S147C(H3C)}$inhA (gray cartoon) showing key TCR-pHLA interface contacts between wildtype HLA-E-inhA, HLA-E$_{S147C(H3C)}$inhA (shown in green, and pink sticks, respectively).

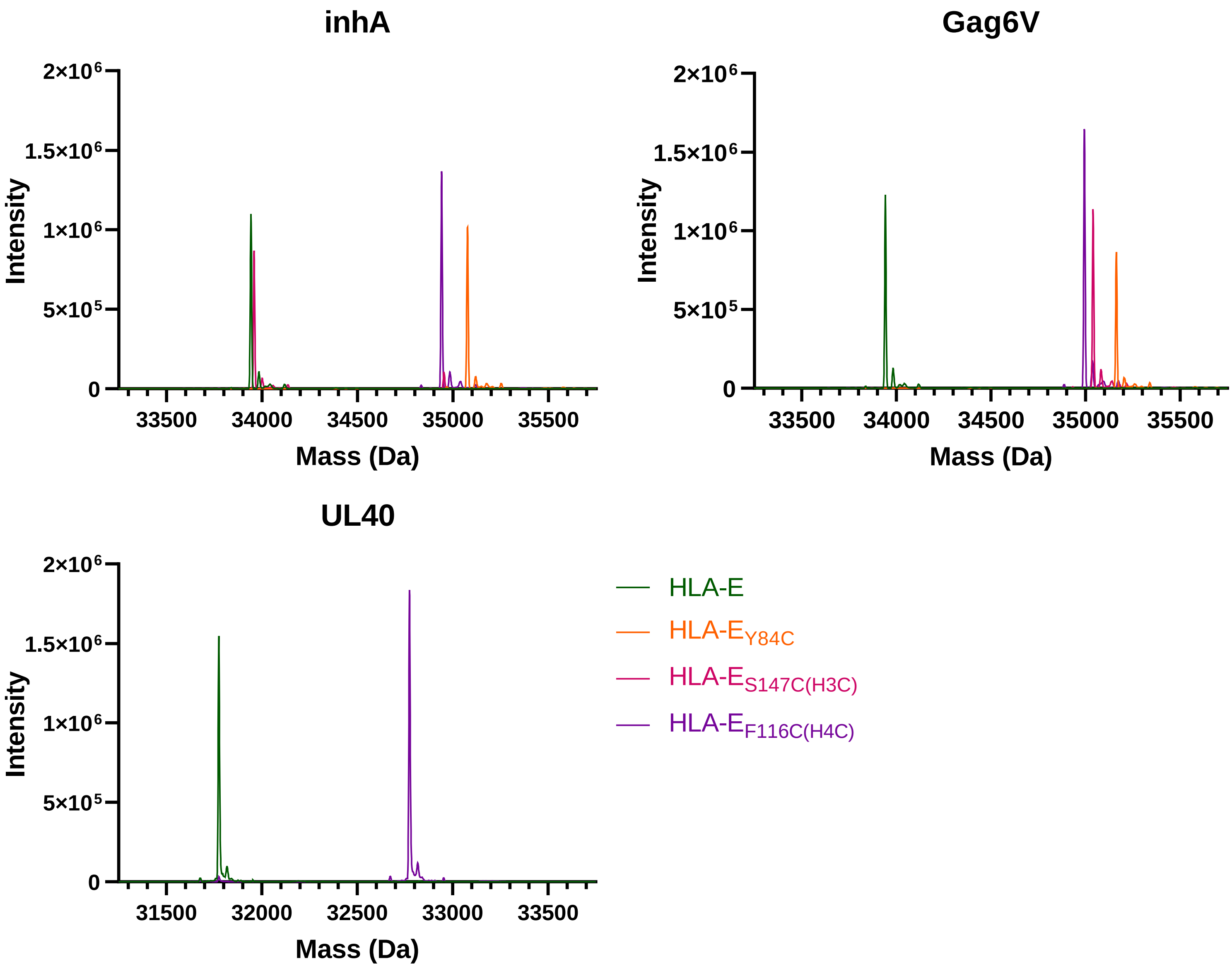

Supplementary Figure S7. Mass spectrometry confirmation of disulfide bond formation between cysteine containing HLA-E mutants and modified peptides.

Intact mass analysis of wildtype HLA-E (green – biotinylated 33941 Da, untagged 31775 Da), HLA-E$_{Y84C}$ (orange – biotinylated 33881 Da), HLA-E$_{S147C(H3C)}$peptide (pink – biotinylated 33957 Da) and HLA-E$_{F116C(H4C)}$peptide (purple - biotinylated 33897 Da, untagged 31731 Da) with inhA, Gag6V and UL40. InhA (978 Da), inhA$_{GCG}$ (1195 Da), H3C inhA (996 Da), and H4C inhA (1010 Da) with biotinylated HLA-E constructs. The observed masses seen for HLA-E$_{Y84C}$ (35076 Da) and HLA-E$_{F116C(H4C)}$peptide (34941 Da) suggest there is disulfide bond formation, however the observed mass for HLA-E$_{S147C(H3C)}$peptide (33959 Da) suggests there is no covalent attachment of peptide to heavy chain. Gag6V (1065 Da), Gag6V$_{GCG}$ (1282 Da), H3C Gag6V (1082 Da), and H4C Gag6V (1096 Da) with biotinylated HLA-E constructs, the observed masses suggest that disulfide bonds are formed for each modified HLA-E/peptide pair, (35162 Da, 34994 Da and 35040 Da respectively). UL40 (1013 Da) and H4C UL40 (1045 Da) with untagged HLA-E constructs. The observed mass of 32775 Da suggests the introduced disulfide bond is formed in HLA-E$_{F116C(H4C)}$UL40.

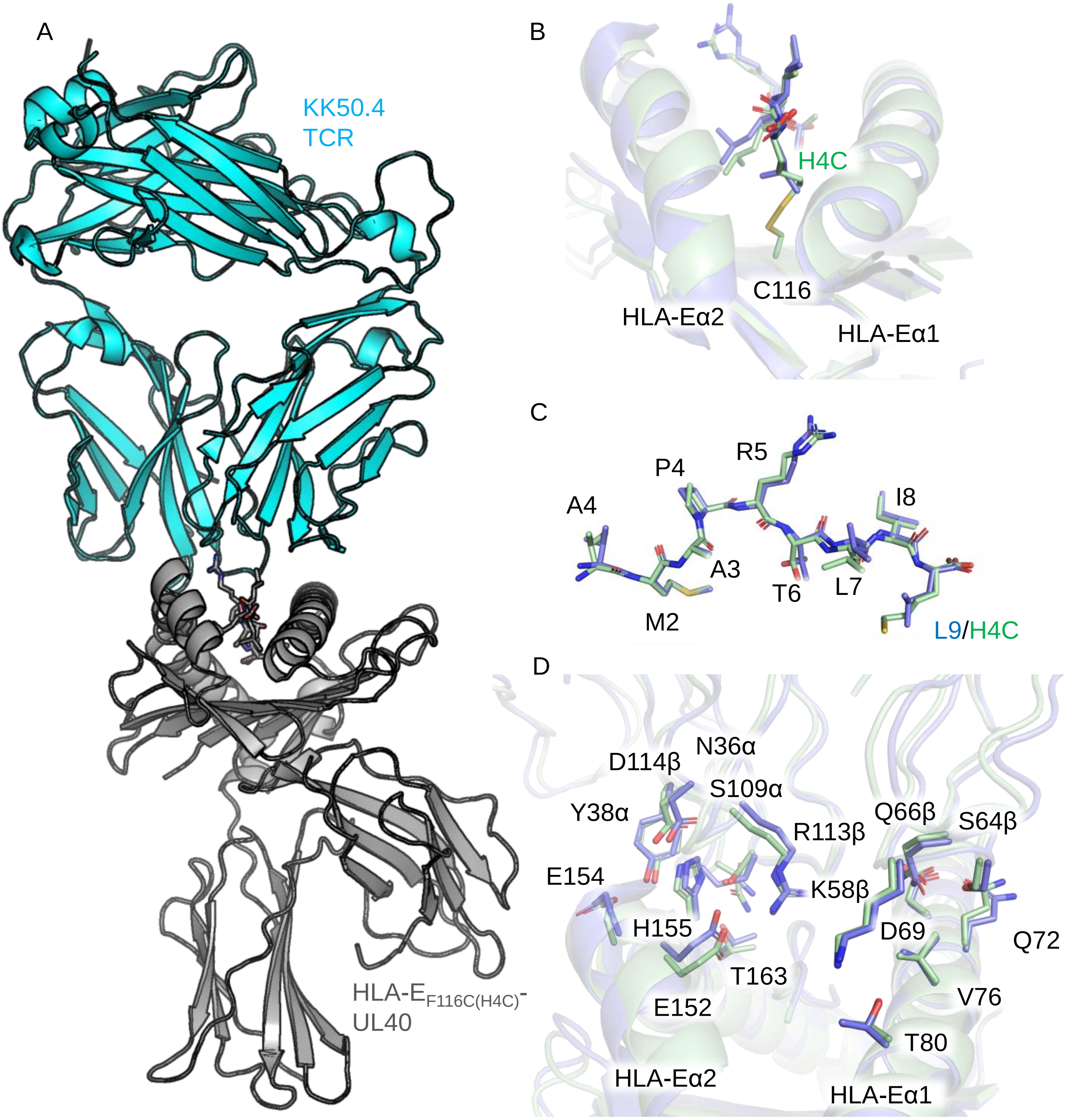

Supplementary Figure S8. Structural comparison of KK50.4 TCR bound to HLA-E-UL40 or HLA-E$_{F116C(H4C)}$UL40.

(**A**) Overall conformation of the KK50.4 TCR (cyan cartoon) in complex with HLA-E$_{F116C(H4C)}$UL40 (gray cartoon and sticks). (**B-D**) Superposition calculated using the HLA-E α-chain, Cα representation. KK50.4-HLA-E-UL40 complex (PDB: 2esv) is colored slate blue and KK50.4-HLA-E$_{F116C(H4C)}$UL40 complex is colored green. (**B**) The disulfide link between the H4C and HLA-E residue F116C is shown, with HLA-E in cartoon and the peptide in sticks. (**C**) Comparison of peptide (sticks) conformation of UL40 in the KK50.4-HLA-E-UL40 complex (PDB: 2esv) and KK50.4-HLA-E$_{F116C(H4C)}$UL40 complex. (**D**) Detailed comparison of the main interactions between the KK50.4-HLA-E-UL40 complex (PDB: 2esv) and KK50.4-HLA-E$_{F116C(H4C)}$UL40 complex. Relevant residues are displayed in stick representation with atomic coloring.
